# Antibacterial and Antibiofilm Potential of Thymol–Benzimidazolium–Chalcone Hybrids Against Clinical MRSA Strains: Insights from Gene Expression Profiling and Molecular Docking

**DOI:** 10.3390/antibiotics15050477

**Published:** 2026-05-08

**Authors:** Salim Yakut, Hakan Ünver, Akın Yiğin, Mehmet Çimentepe, Fadile Yıldız Zeyrek, Özge Öztürk Çimentepe, Metin Yildirim

**Affiliations:** 1 Department of Medical Microbiology, Faculty of Medicine, Harran University, Sanliurfa TR-63050, Türkiye; salimyakut@harran.edu.tr (S.Y.); fadilezeyrek@harran.edu.tr (F.Y.Z.); 2 Department of Chemistry, Faculty of Science, Eskisehir Technical University, Eskisehir TR-26555, Türkiye; hakanunver@eskisehir.edu.tr; 3 Department of Genetics, Faculty of Veterinary Medicine, Harran University, Sanliurfa TR-63040, Türkiye; akinyigin@harran.edu.tr; 4 Department of Pharmaceutical Microbiology, Faculty of Pharmacy, Harran University, Sanliurfa TR-63300, Türkiye; mehmet.cimentepe@harran.edu.tr; 5 Department of Pharmacology, Faculty of Pharmacy, Harran University, Sanliurfa TR-63300, Türkiye; 6 Department of Biochemistry, Faculty of Pharmacy, Cukurova University, Adana TR-01330, Türkiye

**Keywords:** thymol, benzimidazole, chalcone, MRSA, gene expression, biofilm

## Abstract

**Background/Objectives:** Four novel thymol–benzimidazolium–chalcone hybrids were designed based on a molecular hybridization strategy that integrates bioactive scaffolds known for their antimicrobial and antioxidant properties. This approach aims to enhance biological activity through synergistic effects and multi-target interactions, as supported by previous studies on phenolic and benzimidazole derivatives. The inclusion of both antioxidant and antibacterial evaluations was motivated by the well-established role of oxidative stress in bacterial pathogenicity and resistance mechanisms. **Methods:** Their antibacterial potential was initially screened using the disk diffusion method and subsequently evaluated by determining MIC and MBC values against eight clinical *Staphylococcus aureus* isolates. **Results:** Among the tested compounds, compound **3a** emerged as the most potent derivative, exhibiting MIC values ranging from 0.25 to 1 µg/mL. Morphological analysis confirmed significant disruption of bacterial cell integrity, and further investigation demonstrated strong antibiofilm activity accompanied by downregulation of key biofilm- and resistance-associated genes (*icaA*, *dltB*, and *mepA*). Molecular docking studies were performed against selected target proteins, including 1MWT, 3VSL, 3ZG5 (sortase A), and 2ZCS, which are associated with bacterial cell wall biosynthesis, DNA replication, virulence, and metabolic pathways. Compound **3a** exhibited the highest binding affinity, with a docking score of −11.953 kcal/mol against 2ZCS. **Conclusions:** Overall, these findings highlight the potential of thymol-based benzimidazolium–chalcone hybrids as promising multifunctional agents with combined antibacterial, antibiofilm, and antioxidant properties.

## 1. Introduction

Antimicrobial resistance (AMR) represents an escalating global threat. It arises when antibacterial, antifungal, and antiparasitic agents lose their effectiveness against microorganisms such as bacteria, fungi, and parasites. As a result, infections caused by pathogenic microbes become increasingly difficult—and in some cases impossible—to treat, leading to severe clinical consequences. According to the World Health Organization (WHO), bacterial AMR was directly associated with approximately 1.14 million deaths in 2021. Beyond its public health impact, AMR imposes a substantial economic burden, with global costs estimated to reach billions of dollars [1].

*Staphylococcus aureus* (*S. aureus*) is a commensal bacterium commonly found on the skin or in the nasal passages of approximately one in three individuals and typically does not cause disease in healthy hosts [2]. However, methicillin-resistant *Staphylococcus aureus* (MRSA) exhibits resistance to β-lactam antibiotics, including penicillins (such as methicillin and oxacillin) and cephalosporins. Unlike resistance mediated by β-lactamase (penicillinase) production, methicillin resistance in *S. aureus* is primarily associated with alterations in penicillin-binding proteins (PBPs), which reduce antibiotic binding affinity [3]. Due to its multidrug-resistant nature, MRSA significantly limits available therapeutic options, highlighting the urgent need for alternative treatment strategies.

Benzimidazole derivatives exhibit a wide spectrum of biological activities. These compounds include clinically and commercially important agents such as carbendazim and veliparib with anticancer activity, as well as the antiulcer drugs omeprazole and pantoprazole [4]. In addition, benzimidazole-based structures have been reported to possess antibacterial and antiparasitic properties [5]. Several derivatives, including flubendazole, cambendazole, and thiabendazole, have also been demonstrated to exhibit potent anthelmintic activity [6].

Chalcone is a simple molecular scaffold consisting of two aryl rings linked by an α,β-unsaturated carbonyl system (enone) containing an aromatic ketone moiety [7]. It serves as a key biosynthetic precursor in the formation of flavonoids and isoflavonoids. Chalcones have attracted considerable scientific interest due to their broad spectrum of biological activities, including antimicrobial, anticancer, antioxidant, and antileishmanial effects [8]. Clinically used agents such as sofalcone and metochalcone are based on the chalcone framework. The investigations of different hybrid derivatives have revealed their significant potential in the discovery of antimicrobial agents targeting bacterial pathogens [9,10].

Thymol is a phenolic compound found in *Thymus vulgaris* L. and exhibits antibacterial activity against Gram-positive bacteria such as *S. aureus*, as well as Gram-negative species including *Escherichia coli*, *Bacillus thuringiensis*, *Pseudomonas fluorescens*, *Serratia marcescens*, and *Pseudomonas aeruginosa*. In addition to its antimicrobial properties, thymol has been demonstrated to possess anticancer, antioxidant, and anti-inflammatory activities [11,12].

The integration of these bioactive scaffolds may provide synergistic effects, and structural derivatization through various substituents could enhance therapeutic efficacy. Such hybridization strategies may lead to the development of more potent compounds with improved effectiveness against drug-resistant bacterial infections [13]. In this study, thymol–benzimidazolium–chalcone hybrid compounds were synthesized and structurally characterized using FTIR, NMR, and LC–MS/MS analyses. Their antioxidant properties were evaluated, and their antibacterial activity was investigated against clinically isolated and characterized MRSA strains. In addition, antibiofilm activity and the effects of the compounds on gene expression were examined. The experimental findings were further supported through molecular docking studies to elucidate potential mechanisms of action.

## 2. Results

### 2.1. Chemistry

**Figure 1:**
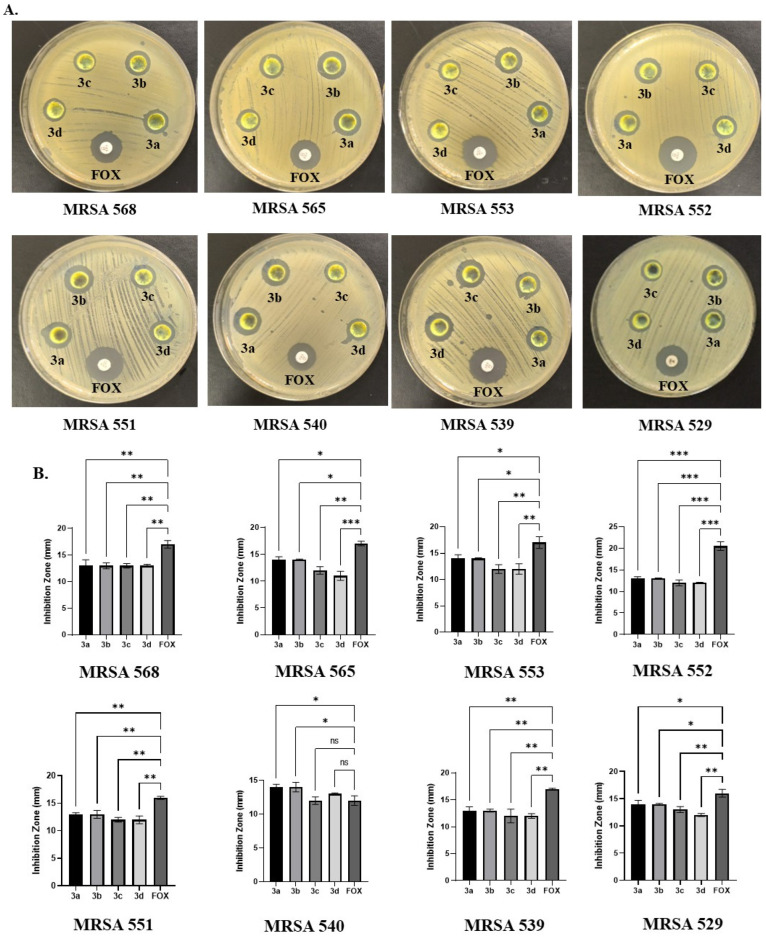
Inhibition zone diameters (mm) of compounds **3a**–**3d** determined by the agar well diffusion method: (**A**) overall inhibition zones, (**B**) antibacterial activity against MRSA. Data are presented as mean ± SD (n = 3). ns indicates not significant (*p* ≥ 0.05); * *p* < 0.05, ** *p* < 0.01, and *** *p* < 0.001 were considered statistically significant.

Figure 1 illustrates the three-step synthetic strategy employed for the preparation of thymol–benzimidazolium–chalcone hybrid derivatives. In the first step, the aldehyde was synthesized via an o-arylation reaction. The second step involved the formation of the chalcone scaffold. In the final step, the benzimidazole nitrogen was quaternized through an N-alkylation reaction. As a result of this multi-step procedure, four novel thymol–benzimidazolium–chalcone hybrid derivatives were successfully obtained, and their structures were thoroughly confirmed using various spectroscopic techniques, including FT-IR, ^1^H and ^13^C NMR, and LC-MS/MS.

### 2.2. Spectroscopic Analysis of the Compounds

Analysis of the FT-IR spectra of compounds **3a**–**d** revealed aromatic C–H stretching vibrations in the range of 2961–3029 cm^−1^. The characteristic carbonyl (C=O) absorptions were observed between 1591 and 1599 cm^−1^, while the olefinic C=C stretching bands appeared in the region of 1501–1555 cm^−1^. These spectral features are consistent with the successful formation of the chalcone moiety within the hybrid structures.

The ^1^H-NMR spectra of compounds **3a**–**d** were analyzed to confirm their structural features. Signals corresponding to the two methyl (-CH_3_) groups of the thymol moiety’s secondary propyl chain were observed at 1.13 ppm, integrating for a total of six protons. The methyl (-CH_3_) group located at the *para*-position of the secondary propyl chain appeared as a singlet at 2.25 ppm, integrating for three protons. In compound **3b**, additional methyl (-CH_3_) proton signals were observed at 2.29 ppm. A multiplet at 3.05 ppm, integrating for one proton, was assigned to the -CH proton of the propyl chain. The methylene (-CH_2_-) bridge present in all four compounds was detected as a singlet around 5.8 ppm. Aromatic protons were observed in the region of 6.80–8.51 ppm, while the -CH protons attached to the benzimidazole ring appeared as singlets around 10.5 ppm. All integration values were consistent with the proposed structures.

The ^13^C-NMR spectra of the compounds were analyzed to confirm their structures. The carbon signal corresponding to the *para*-positioned methyl (-CH_3_) group of the secondary propyl chain on the thymol ring was observed at approximately 20.8 ppm. The two methyl carbons of the secondary propyl group appeared as a single signal at 23.4 ppm, while the CH carbon of the same group was detected at 26.8 ppm. In compound **3b**, the additional methyl carbon was observed at 21.1 ppm. The methylene (-CH_2_-) bridge present in all compounds gave signals in the range of 50.1–50.8 ppm. Aromatic ring carbons were observed between 114.2 and 152.0 ppm, the CH carbons of the benzimidazole ring appeared around 160.9 ppm, and the carbonyl (C=O) carbons were detected at 188.5 ppm. All carbon signals were consistent with the proposed structures.

### 2.3. Antibacterial Activity

#### 2.3.1. Agar Well Diffusion Method

The antibacterial activity of the synthesized compounds **3a**–**3d** was evaluated using the agar well diffusion method against eight clinical MRSA strains. The results revealed that the tested compounds exhibited varying levels of antibacterial activity, as indicated by the inhibition zone diameters (Figure 1). Compounds **3a**–**3d** showed inhibition zone diameters ranging from 11 to 14 mm against the tested clinical strains. The highest antibacterial activity was observed for compounds **3a** and **3b** against MRSA 565, 553, 540, and 529 strains, where the inhibition zone diameter reached 14 mm. The reference antibiotic, FOX, used as a positive control, produced inhibition zones ranging between 12 and 17 mm against the tested strains. Compounds **3a**, **3b**, and **3d** exhibited larger inhibition zone diameters against the MRSA 539 strain compared to cefoxitin. Based on both MIC and inhibition zone diameter results, compounds **3a** and **3b** demonstrated the strongest antibacterial activity, particularly against the MRSA 540 strain.

#### 2.3.2. Evaluation of MIC and MBC Values Against Clinical MRSA Strains

The MIC values of the synthesized benzimidazole–chalcone–thymol derivatives were determined against eight clinical MRSA strains using the resazurin microtiter assay. As shown in Table 1, compounds **3a** and **3b** exhibited MIC values ranging between 0.25 and 4 µg/mL, with the lowest MIC observed for the MRSA 539 strain. Compound **3c** exhibited potent antibacterial activity, with MIC values ranging from 1 to 4 µg/mL across the tested strains. Similarly, compound **3d** demonstrated notable antibacterial activity, with MIC values ranging between 1 and 8 µg/mL against the MRSA isolates. The MIC values of compounds **3a** and **3b** (except for MRSA 552) were found to be lower than those of vancomycin against the tested MRSA strains. In all strains except MRSA 553, 552, and 540, the MIC value of compound **3d** was lower than that of vancomycin. On the other hand, compound **3c** exhibited MIC values comparable to vancomycin against MRSA 568, 565, and 539 strains. Moreover, the MBC values of the compounds against the tested clinical MRSA isolates ranged from 0.5 to 128 µg/mL. These findings indicate that the compounds exhibit varying degrees of bactericidal activity against the clinical MRSA isolates.

### 2.4. Antibiofilm Activity

**Figure 2:**
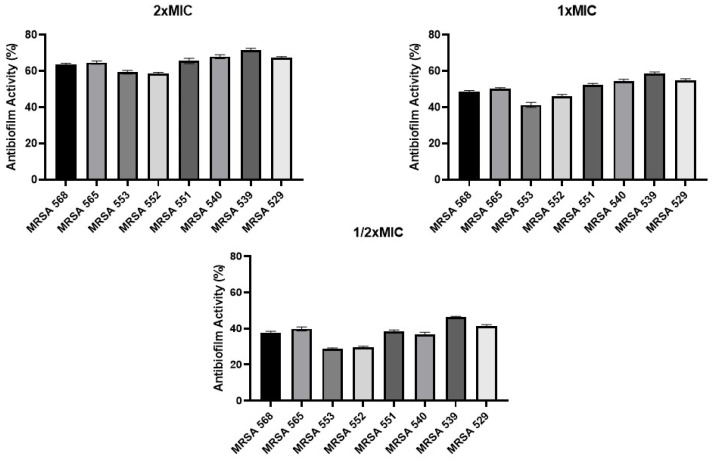
Antibiofilm Activity of **3a** compound.

The antibiofilm activity of the synthesized compound **3a** was evaluated against eight different clinical MRSA strains at three concentrations (2×MIC, 1×MIC, and 1/2×MIC). The results demonstrated that the compound inhibited biofilm formation in a concentration-dependent manner (Figure 2).

At 2×MIC, the compound exhibited strong antibiofilm activity, with inhibition rates ranging approximately between 71.7% and 58.3%, depending on the strain. Among the tested MRSA isolates, compound **3a** displayed the most potent antibiofilm activity against the MRSA 539 strain. At 1×MIC, moderate to high inhibition values were observed, generally between 58.7% and 41.3%. In contrast, at 1/2×MIC, relatively lower antibiofilm activity was detected, with inhibition percentages varying between 46.3% and 28.6% among the tested strains.

Importantly, antibiofilm activity was observed against all eight MRSA strains, although the degree of inhibition varied among isolates. This variability may be associated with strain-specific differences in biofilm-forming capacity and physiological characteristics. Nevertheless, the consistent inhibitory effect across all tested concentrations suggests that the benzimidazole–chalcone–thymol derivative effectively interferes with the biofilm formation process.

Collectively, these findings indicate that the synthesized compound possesses significant antibiofilm potential against MRSA and highlight its possible utility as a promising candidate for the development of novel antimicrobial agents targeting biofilm-associated infections.

### 2.5. Evaluation of Bacterial Morphology

SEM analysis was performed to investigate the antibacterial effects of the selected compound (**3a**) against clinical MRSA strains. SEM observations revealed noticeable alterations in the treated samples compared with the untreated control. Following exposure to the tested compound at 2×MIC, a marked reduction in bacterial cell adhesion was observed.

**Figure 3:**
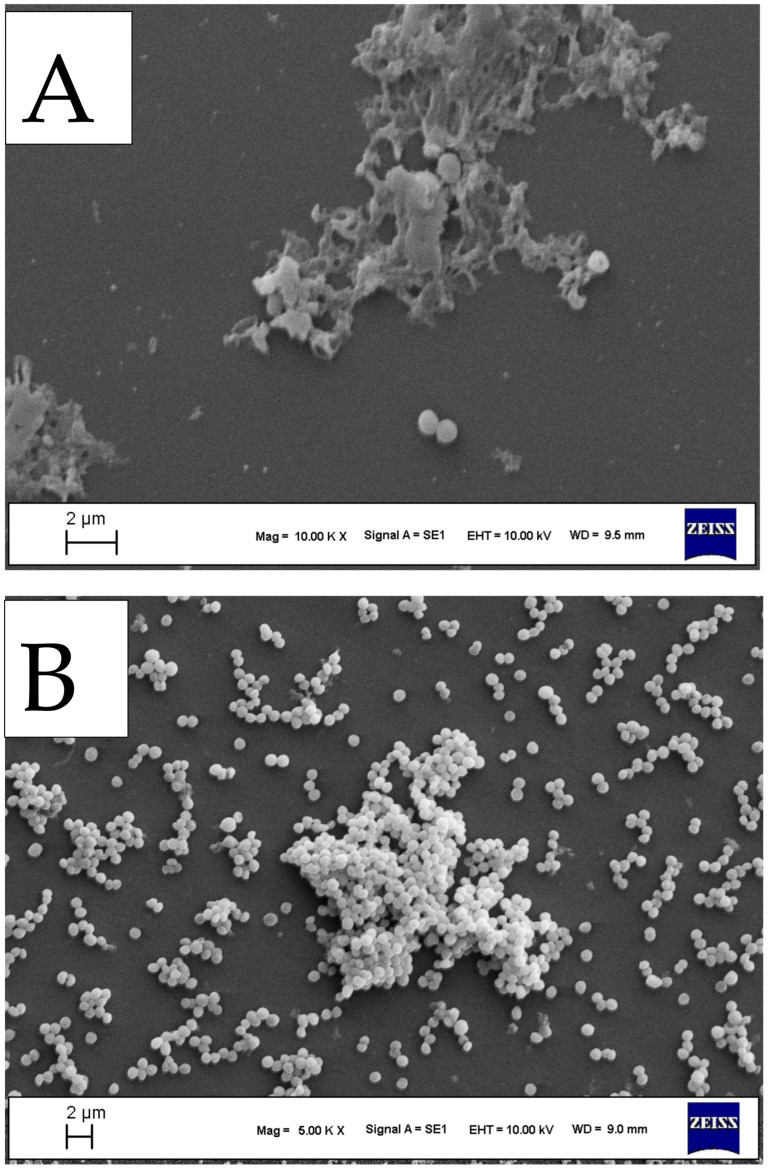
SEM Analyses of the antibacterial activity of compound at 2×MIC against clinical MRSA strain at a magnification of ×10,000 and 5000. (**A**) Treatment with **3a**, (**B**) Untreated MRSA.

In addition, distinct morphological changes in the bacterial cells were detected. The observed antibacterial activity may be attributed to structural alterations in the bacterial cells, which are likely associated with disruption of cell membrane integrity in MRSA following treatment with this compound (Figure 3).

### 2.6. Gene Expression Analysis

**Figure 4:**
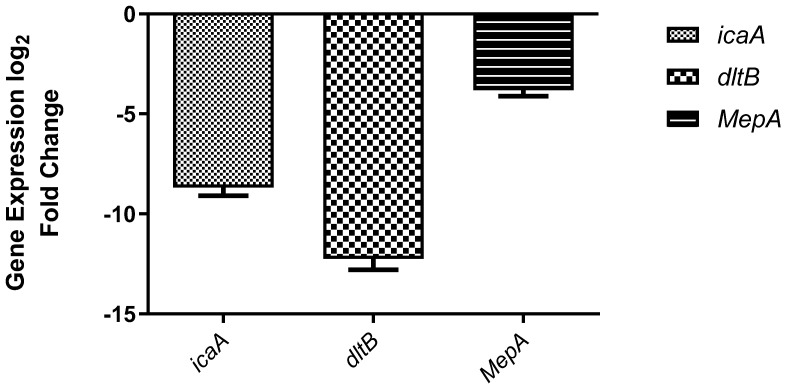
qRT-PCR analysis of icaA, dltB, and mepA gene expression after treatment with compound **3a**. Values represent mean ± SD (n = 3).

Gene expression analysis was performed to evaluate the molecular effects of the most active synthesized compound (**3a**) on key genes associated with antibacterial and antibiofilm activity. The expression levels of *icaA*, *dltB*, and *mepA* were quantified following treatment. The results revealed that compound **3a** significantly downregulated the expression of all three target genes compared to the untreated control. Among them, the most pronounced reduction was observed in the expression of the *dltB* gene (Figure 4). The observed downregulation of *icaA*, *dltB*, and *mepA* genes suggests that compound **3a** exerts its antibacterial and antibiofilm effects through multiple molecular mechanisms. The suppression of *icaA*, which is involved in polysaccharide intercellular adhesion (PIA) synthesis, indicates a potential inhibition of biofilm formation. Similarly, the downregulation of *mepA*, encoding a multidrug efflux pump, may enhance intracellular accumulation of the compound, thereby increasing its antibacterial efficacy. Notably, the most significant downregulation was detected in *dltB*, a gene responsible for D-alanylation of teichoic acids, which plays a crucial role in cell wall charge modification and resistance to antimicrobial agents. This strong suppression suggests that compound **3a** may disrupt cell wall integrity and increase bacterial susceptibility, contributing to its potent antibacterial activity.

### 2.7. Antioxidant Assays

The antioxidant activities of compounds **3a**–**3d** were evaluated by monitoring their ability to scavenge DPPH and ABTS radical species at various concentrations over a defined incubation period. The radical scavenging capacity of each compound was quantified by calculating the IC_50_ values.

According to the DPPH and ABTS radical scavenging assay, the antioxidant activities of the investigated compounds exhibited notable differences. The IC_50_ values for compounds **3a**, **3b**, **3c**, and **3d** were calculated as 73.1 ± 0.34, 148.1 ± 0.67, 97.5 ± 0.54, and 140 ± 0.71, respectively. Similarly, in the ABTS radical scavenging assay, the IC_50_ values were determined to be 67.1 ± 0.72, 27.5 ± 0.43, 92.8 ± 0.82, and 73.4 ± 0.7 µg/mL for the same compounds (Table 2). Based on these findings, compound **3a** and **3b**, which possesses the lowest IC_50_ value, demonstrated the highest radical scavenging activity. These results are consistent with the IC_50_ data and further confirm the superior antioxidant potential of compound **3a** for DPPH and **3b** for ABTS compared to the other tested compounds.

### 2.8. Molecular Docking Studies

**Figure 5:**
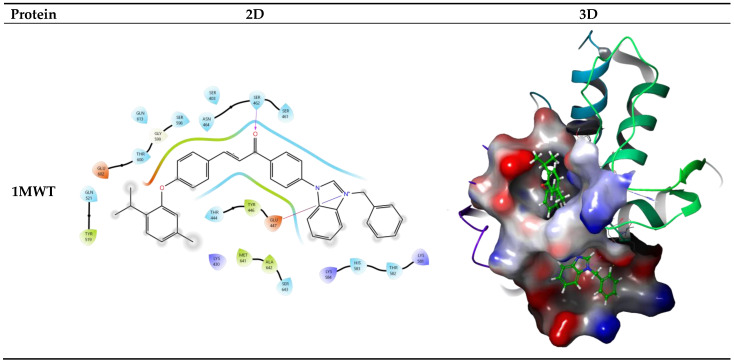

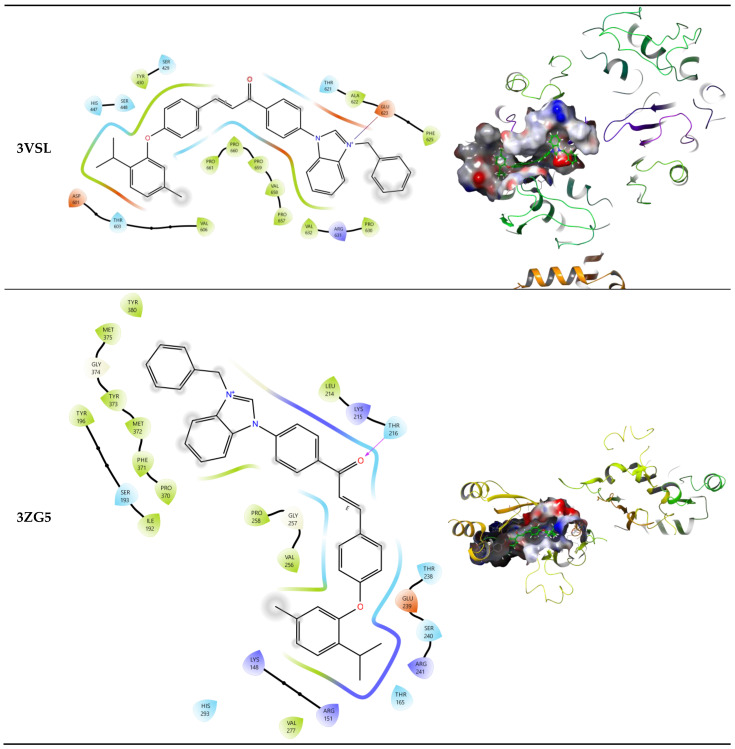

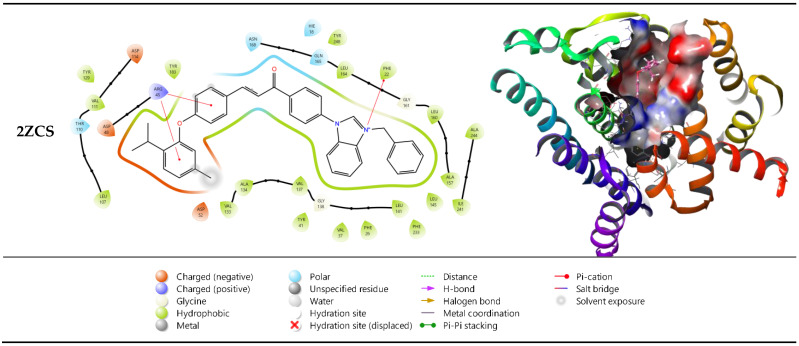
2D and 3D representations of the binding interactions of compound **3a** in the active sites of selected bacterial target proteins.

To support the experimental findings of the most biologically active synthesized compound, in silico molecular docking studies were performed. In this context, docking scores, Glide XP scoring functions, and MM-GBSA binding free energy values were calculated. Additionally, ligand–protein interactions were analyzed in both two-dimensional (2D) and three-dimensional (3D) formats. The selected target proteins (1MWT, 3VSL, 3ZG5, and 2ZCS) correspond to functionally important proteins involved in bacterial cell wall biosynthesis, DNA replication, virulence regulation, and metabolic pathways. These targets were selected based on extensive literature evidence supporting their roles in *Staphylococcus aureus* pathogenicity and antibiotic resistance, as well as their frequent use in previous molecular docking and drug discovery studies. Therefore, their inclusion provides a biologically relevant framework for evaluating the multi-target antibacterial potential of the synthesized compounds. The calculated docking scores ranged between −11.953 and −5.310 kcal/mol. Among the tested compounds, compound **3a** exhibited the strongest binding affinity toward the 2ZCS protein with a docking score of −11.953 kcal/mol. Furthermore, it showed a significant interaction with the 3ZG5 protein, with a docking score of −7.043 kcal/mol. MM-GBSA calculations revealed binding free energy values of −58.77 kcal/mol for 2ZCS and −66.20 kcal/mol for 3ZG5 (Table 3). These results indicate that the ligand–protein complexes are thermodynamically stable. Notably, the more negative MM-GBSA value for the 3ZG5 complex suggests a more stable binding interaction compared to 2ZCS. This observation highlights that binding stability is influenced not only by docking scores but also by solvation effects and free energy contributions. Interaction analysis demonstrated that, in the 2ZCS complex, the aromatic rings of compound **3a** formed π–cation interactions with ARG45. Additionally, the positively charged nitrogen atom (N^+^) of the benzimidazole moiety established a π–cation interaction with PHE22, contributing to binding affinity. In the case of the 3ZG5 protein, the carbonyl group of the ligand formed a hydrogen bond with THR216 (Figure 5).

Overall, these findings indicate that compound **3a** forms strong and specific interactions with the target proteins, providing significant in silico evidence supporting the experimentally observed biological activity.

## 3. Discussion

The benzimidazole ring has been widely studied in medicinal chemistry since the discovery of 5,6-dimethylbenzimidazole as a degradation product of vitamin B12 in 1944 [14,15]. Its importance is largely attributed to its structural similarity to purine bases in DNA, such as adenine and guanine. Structurally, benzimidazole consists of a bicyclic system formed by the fusion of an imidazole ring containing two nitrogen atoms with a benzene ring. Numerous studies have demonstrated that benzimidazole derivatives possess diverse biological activities, including antibacterial, antifungal, antiviral, antileishmanial, and antimalarial effects [16,17,18,19,20].

Bistrović et al. developed new benzimidazole-based scaffolds by introducing phenyl substituents at the 2,5-positions of the triazole ring. The incorporation of amidino groups contributed to derivatives with notable antibacterial properties. Among them, compounds **6**–**8a**–**e** showed broad-spectrum antibacterial activity against MRSA with MIC values of 8–256 µg/mL, comparable to the reference drug ampicillin (MIC = 4 µg/mL) [21]. Rashdan et al. reported that benzimidazole derivatives (**6a**,**b**) exhibited strong antimicrobial effects by targeting DNA gyrase subunit B, thereby inhibiting DNA synthesis and inducing bacterial cell death. These compounds showed inhibition zones of 23–29 mm against *S. aureus*, exceeding that of ciprofloxacin (20 mm) [22]. In our previous study, the lowest MIC value of benzimidazolium salts against MRSA was determined as 15.62 µg/mL [23].

In recent years, medicinal chemistry strategies have increasingly focused on developing therapeutic agents that are highly effective, selective, and associated with minimal toxicity. One widely adopted approach is molecular hybridization, which combines two or more pharmacophoric units within a single molecular framework to generate compounds with improved biological properties and multi-target interactions [24]. Chalcones, originally identified as intermediates in flavonoid biosynthesis, contain an α,β-unsaturated carbonyl system linking two aromatic rings, which plays a key role in their biological activity [25]. These compounds exhibit diverse pharmacological effects, including antibacterial, antifungal, anti-inflammatory, antioxidant, and anticancer activities [26,27].

Natural compounds such as thymol have also attracted attention as potential sources of therapeutic agents with reduced adverse effects [28]. Thymol, a monoterpene-based natural product, exhibits a wide spectrum of biological activities, including antibacterial, antifungal, and antioxidant effects [29,30]. Due to these properties, thymol-derived structures have been incorporated into hybrid molecules to enhance biological activity.

Padhy et al. reported that benzimidazole–chalcone derivatives exhibited MIC values ranging from 62.5 to 500 µg/mL against *S. aureus* [31]. Similarly, 2-chloroquinoline–benzimidazole chalcone derivatives demonstrated MIC values between 250 and 1000 µg/mL, with inhibition zones of 17.5 ± 0.29 mm to 21 ± 0.00 mm [32].

In the present design, benzimidazole, chalcone, and thymol moieties were rationally combined to exploit their complementary pharmacological properties within a single molecular framework. The benzimidazole core was selected for its well-documented interaction with bacterial targets such as DNA gyrase and its structural similarity to purine bases, enabling interference with nucleic acid processes. The chalcone unit, containing an α,β-unsaturated carbonyl system, was incorporated as a reactive pharmacophore capable of interacting with multiple biological targets, thereby contributing to antibacterial and antibiofilm activity. Thymol was introduced as a natural phenolic fragment to enhance membrane permeability and disrupt bacterial cell integrity due to its lipophilic character.

The linker architecture, based on a benzimidazolium–chalcone conjugation, was designed to maintain electronic conjugation and structural rigidity while allowing optimal spatial orientation of the pharmacophoric units for effective target interaction. This hybridization strategy was expected to produce synergistic effects by combining membrane disruption, enzyme inhibition, and redox-related mechanisms.

In the present study, among the synthesized benzimidazole–chalcone–thymol derivatives, **3a** and **3b** showed superior inhibition zone diameters against the MRSA 540 strain (14 mm) compared to cefoxitin (12 mm). Furthermore, these compounds exhibited significant antibacterial activity against clinical MRSA strains, with notably low MIC values. These findings suggest that the synthesized derivatives may be considered promising antimicrobial candidates for the treatment of MRSA-associated infections; however, further studies are required for clinical application.

Biofilm-associated MRSA represents a clinically significant pathogen capable of causing a wide range of infections, from skin and soft tissue infections to severe conditions such as bloodstream infections, osteomyelitis, and infective endocarditis. These infections pose a major public health concern due to multidrug resistance and high morbidity and mortality rates [33]. One of the key factors contributing to MRSA pathogenicity is its ability to form biofilms, where bacterial cells are embedded within a self-produced extracellular polymeric matrix [34]. Biofilms protect bacteria from environmental stress and antimicrobial agents, reducing antibiotic efficacy.

Efflux pump systems also play a crucial role by actively expelling antimicrobial agents, thereby limiting intracellular drug accumulation and contributing to resistance. Chalcone derivatives have been reported to inhibit the NorA efflux pump in *S. aureus*, suppressing efflux activity within biofilms [35]. Nandwana et al. demonstrated that imidazo/benzimidazoquinazoline derivatives exhibited significant antibacterial and antibiofilm activity, likely through disruption of the biofilm matrix [36]. Similarly, several benzimidazole derivatives (e.g., UM-C42, UM-C162, UM-C164) were shown to inhibit biofilm formation by more than 70% [37]. Nawaz et al. also reported that hydroxychalcone derivatives exhibited significant antibiofilm activity [35].

It has been suggested that hydroxyl and methoxy groups present in chalcone structures play a critical role in inhibiting *S. aureus* biofilm formation. Additionally, chalcones lacking hydroxyl groups in the B ring may exhibit enhanced membrane-disruptive activity, while lipophilic hydroxylated groups in the A ring may increase antibacterial activity by destabilizing membrane structures [38]. Hydroxy-halogenated chalcones have also been reported to exhibit strong antibiofilm activity [39].

Consistent with these findings, in the present study, compound **3a** exhibited dose-dependent antibiofilm activity against clinical MRSA strains. These results suggest that compound **3a** may serve as a promising candidate for preventing or reducing MRSA-associated biofilm formation.

Benzimidazole and chalcone derivatives have been widely studied in medicinal chemistry due to their diverse biological activities, including notable antioxidant properties. Benzimidazole derivatives exhibit significant free radical scavenging activity, as evidenced by low IC_50_ values in DPPH assays, indicating strong antioxidant potential compared to standard antioxidants such as BHT. Likewise, numerous studies on chalcone derivatives have demonstrated that their α,β-unsaturated carbonyl system facilitates effective hydrogen/electron donation, resulting in potent radical scavenging activity in both DPPH and ABTS assays. Therefore, the incorporation of benzimidazole and chalcone pharmacophores into a single molecular framework is expected to enhance radical scavenging efficiency and overall antioxidant capacity [40,41].

The study by Archie et al. assessed the antioxidant properties of selected benzimidazole derivatives, revealing strong activity across all derivatives, with IC_50_ values ranging from 3.17 to 7.59 μg/mL, whereas BHT showed a comparatively higher IC_50_ value of 18.42 μg/mL [42]. Murti et al. reported that benzimidazolyl-chalcones exhibited DPPH antioxidant activity ranging from approximately 39% to 70% [43]. Similarly, Kouakou et al. demonstrated that benzyl-benzimidazolyl-chalcone derivatives showed ABTS radical scavenging activity of 39.61%, 66.09%, and 84.20% [44]. In another study, thymol-based benzimidazole derivatives displayed excellent radical scavenging ability compared to positive controls, with inhibition rates increasing in a dose-dependent manner [45]. In our study, the antioxidant activities of the synthesized compounds **3a**–**3d** were evaluated using DPPH and ABTS radical scavenging assays, and the results were expressed as IC_50_ values. In the DPPH assay, the IC_50_ values were determined to be 73.1 ± 0.34, 148.1 ± 0.67, 97.5 ± 0.54, and 140 ± 0.71 µg/mL for compounds **3a**–**3d**, respectively. Among these, compound **3a** exhibited the strongest activity, as indicated by its lowest IC_50_ value, while compound **3b** showed comparatively weaker activity. In the ABTS assay, the IC_50_ values were found to be 67.1 ± 0.72, 27.5 ± 0.43, 92.8 ± 0.82, and 73.4 ± 0.7 µg/mL for compounds **3a**–**3d**, respectively. Notably, compound **3b** demonstrated the highest antioxidant activity in this assay, suggesting a possible variation in radical scavenging mechanisms between DPPH and ABTS systems. Overall, the results indicate that all tested compounds possess moderate to strong antioxidant potential, with activity profiles depending on both the assay type and structural differences among the compounds. Benzimidazole–chalcone derivatives, due to their lipophilic and phenolic groups, can penetrate bacterial membranes, disrupt membrane integrity, inhibit metabolic enzymes, weaken bacterial oxidative stress response mechanisms, and ultimately contribute to bacterial death by causing damage to DNA and proteins. Specifically, the benzimidazole ring, owing to its heterocyclic nitrogen structure, can interfere with DNA and inhibit bacterial enzyme activity, while the chalcone moiety can increase membrane permeability, exhibiting both antioxidant and pro-oxidant behaviors and thereby exerting a toxic effect on bacterial cells. Therefore, antioxidant activity is closely related to structural and biochemical mechanisms that support the antibacterial effects of such compounds [46,47].

Overall, compounds **3a**–**3d** stand out as dual-functional molecules exhibiting both antioxidant and antibacterial activities, highlighting their potential as promising bioactive agents for pharmaceutical and biomedical applications.

## 4. Materials and Methods

### 4.1. Reagents

All chemicals and solvents were purchased from commercial suppliers and used without additional purification. FT-IR spectra were recorded using a PerkinElmer (Waltham, MA, USA) Spectrum 100 spectrometer equipped with an ATR accessory in the 4000–700 cm^−1^ range. NMR measurements were performed on an Agilent (Agilent Technologies, Inc., Santa Clara, CA, USA) 400 MHz FT-NMR spectrometer, and ^1^H and ^13^C NMR spectra were utilized for structural confirmation and purity assessment. The progress of the reactions was monitored by thin-layer chromatography (TLC). The LC-MS/MS mass spectra of the synthesized thymol–benzimidazolium–chalcone hybrid compounds were obtained using a Shimadzu (Shimadzu Tokyo Innovation Plaza, Tokyo, Japan) LC-MS/MS-8030 series mass spectrometer in the ESI positive ion mode via the SIM technique.

### 4.2. Methods

#### 4.2.1. Synthesis Procedure of Compounds

**4.-(2-.isopropyl-5-methylphenoxy)benzaldehyde (Compound 1)**

Initially, 1 equivalent of thymol (1.0 g) and 2 equivalents of potassium carbonate (1.8 g) were stirred in 10 mL of dimethyl sulfoxide (DMSO) at room temperature for 10 min. Subsequently, 1 equivalent of 4-fluorobenzaldehyde (0.8 g) was added to the reaction mixture, and the mixture was heated at 120 °C for 24 h. The progress of the reaction was monitored by thin-layer chromatography (TLC). Upon completion of the reaction, the solvent was evaporated under reduced pressure, and the resulting crude product was purified by column chromatography using ethyl acetate/hexane (1:9, *v*/*v*) as the eluent. Compound **1** was obtained as a liquid.

**Yield: 81%. ^1^H-NMR (400 MHz, DMSO-*d*_6_) *δ* (ppm):** 9.88 (s, 1H), 7.88 (d, *J* = 6.5 Hz, 2H), 7.31 (d, *J* = 6.0 Hz, 1H), 7.07 (d, *J* = 7.9 Hz, 1H), 7.01 (d, *J* = 8.7 Hz, 2H), 6.83 (s, 1H), 2.98 (p, *J* = 7.4 Hz, 1H), 2.25 (s, 3H), 1.10 (d, *J* = 7.3 Hz, 6H). **^13^C-NMR (100 MHz, DMSO-*d_6_*) *δ* (ppm):** 191.83, 163.66, 151.41, 137.65, 137.45, 132.51, 131.20, 127.71, 127.09, 121.96, 116.81, 26.81, 23.36, 20.83.

**1.-(4-(1H-benzo[d]imidazol-1-yl)phenyl)-3-(4-(2-isopropyl-5-methylphenoxy) phenyl)prop-2-en-1-one (Compound 2)**

For the synthesis of Compound **2**, 1 equivalent of Compound **1** (1.0 g) and 1 equivalent of the ketone (0.9 g) were mixed in 20 mL of ethanol. Subsequently, 1.5 equivalents of potassium hydroxide (0.3 g dissolved in ethanol) were added dropwise to the reaction mixture at room temperature. Upon completion of the reaction, the resulting precipitate was collected by filtration and washed with cold ethanol. The precipitate was dried and used directly in the subsequent step without further purification.

Yield: 75%. **^1^H-NMR (400 MHz, DMSO-*d*_6_) *δ* (ppm):** 8.71 (s, 1H), 8.38 (d, *J* = 6.7 Hz, 2H), 7.91 (d, *J* = 8.2 Hz, 5H), 7.83–7.72 (m, 3H), 7.35 (dq, *J* = 16.8, 8.2, 7.8 Hz, 3H), 7.04 (d, *J* = 7.9 Hz, 1H), 6.94 (d, *J* = 7.1 Hz, 2H), 6.80 (s, 1H), 3.05 (p, *J* = 7.0 Hz, 1H), 2.25 (s, 3H), 1.13 (d, *J* = 6.8 Hz, 6H). **^13^C-NMR (100 MHz, DMSO-*d*_6_) *δ* (ppm):** 188.38, 160.76, 152.10, 144.51, 144.41, 143.74, 140.12, 137.44, 137.26, 136.79, 133.04, 131.61, 130.93, 129.33, 127.56, 126.54, 124.29, 123.70, 123.33, 121.48, 120.76, 120.60, 117.23, 111.42, 26.82, 23.40, 20.88.

**Thymol–benzimidazoium–chalcone hybrid derivatives**

Thymol–benzimidazolium–chalcone hybrid derivatives (**3a**–**d**) were synthesized via N-alkylation (quaternization) of the benzimidazole nitrogen atom, yielding N-quaternized benzimidazolium bromide salts. Accordingly, Compound **2** (0.1 g, 1.0 equiv) reacted separately with benzyl bromide (0.03 g), 4-methylbenzyl bromide (0.04g), 4-(trifluoromethyl)benzyl bromide (0.05 g), or 4-bromobenzyl bromide (0.05 g) (1.0 equiv) in acetonitrile. The reactions were conducted at 70 °C under magnetic stirring for 48 h. The resulting cationic benzimidazolium bromide salts precipitated from the reaction medium and were isolated by filtration, washed with diethyl ether, and dried.

**3.-benzyl-1-(4-(3-(4-(2-isopropyl-5-methylphenoxy)phenyl)acryloyl)phenyl)-1H-benzo[d]imidazol-3-ium bromide (Compound 3a)** **:**

White solid, Yield: 78%, 0.101 g.

**FT-IR (KBr, cm^−1^):** 3017 (aromatic CH), 1599 (carbonyl C=O), 1550 (olefin C=C).

**^1^H-NMR (400 MHz, DMSO-*d*_6_) *δ* (ppm):** 10.55 (s, 1H), 8.50 (d, *J* = 8.2 Hz, 2H), 8.09 (d, *J* = 8.2 Hz, 2H), 8.05–8.01 (m, 1H), 7.94 (d, *J* = 7.7 Hz, 4H), 7.81 (d, *J* = 15.5 Hz, 1H), 7.72 (dd, *J* = 6.4, 3.0 Hz, 2H), 7.66 (d, *J* = 7.4 Hz, 2H), 7.42 (dt, *J* = 12.7, 7.2 Hz, 3H), 7.31 (d, *J* = 8.0 Hz, 1H), 7.05 (d, *J* = 7.9 Hz, 1H), 6.95 (d, *J* = 8.3 Hz, 2H), 6.80 (s, 1H), 5.89 (s, 2H), 3.05 (p, *J* = 7.0 Hz, 1H), 2.25 (s, 3H), 1.13 (d, *J* = 6.9 Hz, 6H). **^13^C-NMR (100 MHz, DMSO-*d_6_*) *δ* (ppm):** 188.55, 160.93, 152.07, 145.07, 143.59, 139.34, 137.46, 137.27, 137.02, 134.04, 131.76, 131.57, 131.39, 130.88, 129.40, 129.26, 129.22, 128.98, 128.13, 127.66, 127.60, 126.60, 126.03, 121.50, 120.68, 117.25, 114.80, 114.31, 50.81, 26.84, 23.40, 20.89.

ESIMS: 563.75.

**1.-(4-(3-(4-(2-isopropyl-5-methylphenoxy)phenyl)acryloyl)phenyl)-3-(4-methylbenzyl)-1H-benzo[d]imidazol-3-ium bromide (Compound 3b)** **:**

White solid, Yield: 71%, 0.099 g.

**FT-IR (KBr, cm^−1^):** 3029 (aromatic CH), 1592 (carbonyl C=O), 1554 (olefin C=C).

**^1^H-NMR (400 MHz, DMSO-*d*_6_) *δ* (ppm):** 10.47 (s, 1H), 8.49 (d, *J* = 8.1 Hz, 2H), 8.05 (dd, *J* = 22.9, 7.6 Hz, 3H), 7.93 (d, *J* = 8.2 Hz, 4H), 7.81 (d, *J* = 15.4 Hz, 1H), 7.71 (d, *J* = 6.8 Hz, 2H), 7.54 (d, *J* = 7.7 Hz, 2H), 7.31 (d, *J* = 7.4 Hz, 1H), 7.23 (d, *J* = 7.7 Hz, 2H), 7.05 (d, *J* = 8.0 Hz, 1H), 6.95 (d, *J* = 8.3 Hz, 2H), 6.80 (s, 1H), 5.82 (s, 2H), 3.05 (dt, *J* = 13.1, 6.7 Hz, 1H), 2.27 (d, *J* = 13.8 Hz, 6H), 1.13 (d, *J* = 6.7 Hz, 6H). **^13^C-NMR (100 MHz, DMSO-*d_6_*) *δ* (ppm):** 188.53, 160.94, 152.05, 145.08, 143.46, 139.33, 138.74, 137.46, 137.27, 137.02, 131.75, 131.56, 131.33, 131.00, 130.87, 129.93, 129.21, 129.01, 128.11, 127.60, 126.60, 126.02, 121.50, 120.65, 117.25, 114.83, 114.29, 50.67, 26.83, 23.41, 21.19, 20.89.

ESIMS: 577.75.

**1.-(4-(3-(4-(2-isopropyl-5-methylphenoxy)phenyl)acryloyl)phenyl)-3-(4-(trifluoromethyl)benzyl)-1H-benzo[d]imidazol-3-ium bromide (Compound 3c)** **:**

White solid, Yield: 65%, 0.097 g.

**FT-IR (KBr, cm^−1^):** 2961 (aromatic CH), 1594 (carbonyl C=O), 1501 (olefin C=C).

**^1^H-NMR (400 MHz, DMSO-*d*_6_) *δ* (ppm):** 10.52 (s, 1H), 8.50 (d, *J* = 8.2 Hz, 2H), 8.08 (d, *J* = 8.2 Hz, 2H), 8.03 (dd, *J* = 6.6, 3.0 Hz, 1H), 7.95 (dd, *J* = 13.0, 5.5 Hz, 4H), 7.89–7.78 (m, 5H), 7.77–7.71 (m, 2H), 7.31 (d, *J* = 7.9 Hz, 1H), 7.05 (d, *J* = 7.9 Hz, 1H), 6.95 (d, *J* = 8.3 Hz, 2H), 6.80 (s, 1H), 6.00 (s, 2H), 3.05 (p, *J* = 7.1 Hz, 1H), 2.25 (s, 3H), 1.13 (d, *J* = 6.9 Hz, 6H).

**^13^C-NMR (100 MHz, DMSO-*d*_6_) *δ* (ppm):** 188.54, 160.94, 152.06, 145.08, 143.94, 139.38, 138.68, 137.46, 137.27, 137.00, 131.75, 131.56, 131.39, 130.90, 129.85, 129.80, 129.53, 129.21, 128.20, 127.79, 127.60, 126.60, 126.27, 126.23, 126.19, 126.01, 125.85, 123.14, 121.49, 120.68, 117.25, 114.65, 114.35, 50.13, 26.84, 23.40, 20.89.

ESIMS: 631.70.

**3.-(4-bromobenzyl)-1-(4-(3-(4-(2-isopropyl-5-methylphenoxy)phenyl)acryloyl)phenyl)-1H-benzo[d]imidazol-3-ium bromide (Compound 3d)** **:**

White solid, Yield: 61%, 0.092 g.

**FT-IR (KBr, cm^−1^):** 3025 (aromatic CH), 1591 (carbonyl C=O), 1555 (olefin C=C).

**^1^H-NMR (400 MHz, DMSO-*d*_6_) *δ* (ppm):** 10.45 (s, 1H), 8.49 (d, *J* = 8.2 Hz, 2H), 8.10–8.04 (m, 2H), 8.01 (d, *J* = 4.7 Hz, 1H), 7.93 (d, *J* = 8.5 Hz, 4H), 7.81 (d, *J* = 15.4 Hz, 1H), 7.73 (d, *J* = 6.9 Hz, 2H), 7.62 (t, *J* = 7.7 Hz, 4H), 7.31 (d, *J* = 7.9 Hz, 1H), 7.05 (d, *J* = 7.9 Hz, 1H), 6.95 (d, *J* = 8.2 Hz, 2H), 6.80 (s, 1H), 5.85 (s, 2H), 3.09–3.01 (m, 1H), 2.25 (s, 3H), 1.13 (d, *J* = 6.9 Hz, 6H). **^13^C-NMR (100 MHz, DMSO-*d*_6_) *δ* (ppm):** 188.54, 160.94, 152.06, 145.08, 143.72, 139.37, 137.46, 137.27, 136.99, 133.38, 132.30, 131.75, 131.56, 131.29, 130.88, 129.21, 128.17, 127.72, 127.60, 126.60, 126.00, 122.68, 121.49, 120.67, 117.26, 114.71, 114.32, 50.12, 26.84, 23.40, 20.89.

ESIMS: 642.60.

**Figure 6:**
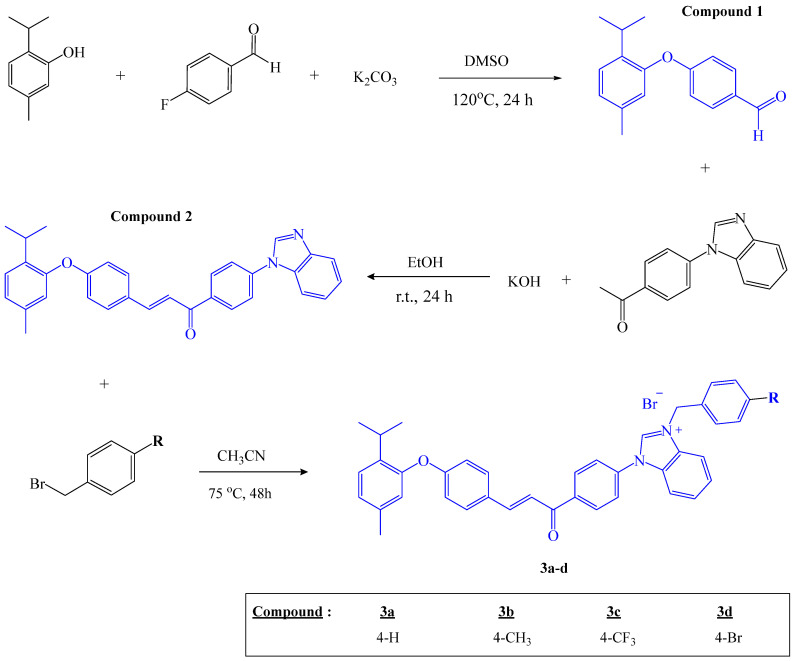
Synthesis procedure of thymol–benzimidazoium–chalcone hybrid derivatives.

**Figure 7:**
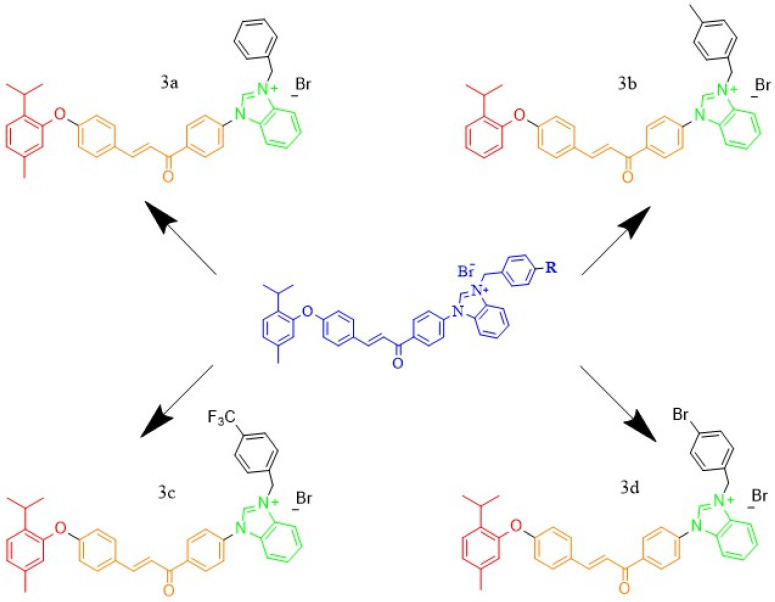
Structural representation of the synthesized benzimidazolium–chalcone derivatives (**3a**–**3d**) showing substituent-dependent variations.

The FT-IR, 1H-NMR, 13C-NMR, and ESIMS spectra are provided in the Supplementary File. The syntheses scheme and synthesized of thymol–benzimidazoium–chalcone hybrid compounds is given in Figure 6 and Figure 7. 

#### 4.2.2. Identification of Clinical *Staphylococcus aureus* Isolates by MALDI-TOF MS

**Figure 8:**
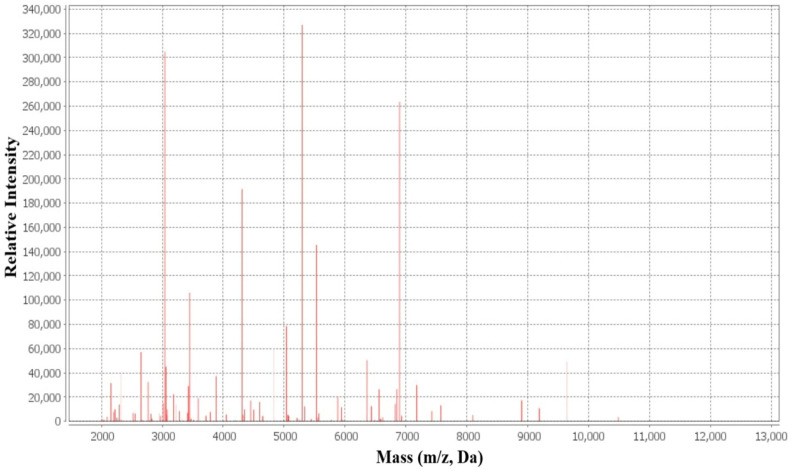
Identification of clinical *S. aureus* isolates by MALDI-TOF MS based on characteristic *m*/*z* spectral profiles.

Species-level identification of the clinical isolates was performed using matrix-assisted laser desorption/ionization time-of-flight mass spectrometry (MALDI-TOF MS) with the VITEK MS system (bioMérieux, Marcy-l’Etoile, France). Prior to analysis, the instrument was calibrated using the *Escherichia coli* test standard provided by the manufacturer. For quality assurance and to verify identification accuracy, the reference strain *Staphylococcus aureus* ATCC 29213 was included as a quality control strain and analyzed concurrently with the clinical isolates (Figure 8).

#### 4.2.3. Phenotypic Determination of MRSA and MSSA Isolates

Methicillin susceptibility of the isolates identified as *S. aureus* at the species level by MALDI-TOF MS was determined using the disk diffusion method. The interpretation of susceptibility results was performed according to the current clinical breakpoints published by the European Committee on Antimicrobial Susceptibility Testing (EUCAST) [48].

#### 4.2.4. Determination of MRSA by Disk Diffusion Method

Pure colonies were suspended in sterile saline and adjusted to a turbidity equivalent to the 0.5 McFarland standard. The bacterial suspension was inoculated onto the surface of Mueller–Hinton agar plates using a sterile swab and a three-directional streaking technique to obtain a uniform lawn of growth. Subsequently, 30 µg cefoxitin disks were placed on the agar surface, and the plates were incubated at 35 ± 1 °C for 16–18 h. Following incubation, inhibition zone diameters were measured in millimeters and interpreted according to EUCAST guidelines [48].

#### 4.2.5. Agar Well Diffusion Assay

The in vitro antimicrobial activity of the tested compounds was evaluated using the agar well diffusion method [49]. Bacterial strains were obtained from fresh cultures incubated for 18–24 h and suspended in sterile 0.85% NaCl solution. The turbidity was adjusted to the 0.5 McFarland standard (approximately 1–2 × 10^8^ CFU/mL). The suspensions were uniformly inoculated onto Mueller–Hinton agar plates using a sterile cotton swab with a three-directional streaking technique. Plates were left at room temperature for 10–15 min to allow absorption of excess moisture. Wells of 7 mm diameter were aseptically created using a sterile perforator, and the agar plugs were removed. Subsequently, 100 µL of stock compound solution (4 mg/mL) was added to each well. A 30 µg cefoxitin (FOX) disc (Oxoid, UK) was used as a positive control. Plates were incubated at 37 °C for 18–24 h. Inhibition zone diameters were measured in millimeters (mm), and mean values were calculated.

#### 4.2.6. Determination of Minimum Inhibitory Concentration (MIC) and Minimum Bactericidal Concentration (MBC)

The MIC values of compounds **3a**–**3d** against eight different clinical MRSA isolates were determined using the resazurin-based microdilution method [50]. Sterile U-bottom 96-well microplates were used. A total of 100 µL of cation-adjusted Mueller–Hinton broth (CAMHB) was added to each well. Pre-dilutions of compounds **3a** and **3b** were prepared from stock solutions at a ratio of 1/32, and 100 µL was transferred to the first well. Serial dilutions were performed up to the tenth well. The final concentration range was 0.06–32 µg/mL for compounds **3a** and **3b**, and 0.25–128 µg/mL for compounds **3c** and **3d**.

The eleventh well served as the negative control (CAMHB + compound), and the twelfth well as the positive control (CAMHB + bacterial suspension). A bacterial suspension adjusted to 0.5 McFarland standard (approximately 1 × 10^8^ CFU/mL) was further diluted 1:100. Subsequently, 100 µL of the bacterial suspension was added to each well (except the negative control), resulting in a final concentration of 5 × 10^5^ CFU/mL. The microplates were incubated at 37 °C for 18–24 h.

After incubation, 20 µL of 0.01% (*w*/*v*) sterile resazurin solution was added to each well, and the plates were re-incubated at 37 °C for 3 h. The color change was visually evaluated, and the lowest compound concentration at which the blue color was maintained was considered the MIC [51]. All experiments were performed in duplicate.

For MBC determination, 10 µL samples were taken from wells showing no visible growth in the MIC assay and spread onto MHA plates. The plates were incubated at 37 °C for 18–24 h. After incubation, colony formation was evaluated, and the lowest compound concentration yielding 3–9 CFU/mL was recorded as the minimum bactericidal concentration (MBC).

#### 4.2.7. Antibiofilm Activity

Based on antibacterial screening results, compound **3a**, which exhibited the lowest MIC values, was further evaluated for antibiofilm activity against clinical MRSA isolates using the crystal violet staining method [52].

MRSA isolates were cultured on blood agar plates, and suspensions were prepared in sterile saline to match the 0.5 McFarland standard. The suspensions were diluted with TSB, and 200 µL was added to each well of sterile 96-well polystyrene microplates. Plates were incubated at 37 °C for 24 h under static conditions to allow biofilm formation.

Compound **3a** was added at concentrations of 2×MIC, 1×MIC, and 1/2×MIC, followed by incubation for another 24 h. Wells without compound served as biofilm controls. After incubation, planktonic cells were removed, and wells were washed with PBS. Biofilms were fixed with methanol for 10 min and stained with 0.5% crystal violet for 15 min. Excess stain was washed off, and plates were air-dried. The dye was solubilized with 33% acetic acid, and optical density was measured at 570 nm. Antibiofilm activity was calculated as:

Antibiofilm rate (%) = (C − S)/C × 100

where C is the control absorbance and S is the sample absorbance.

#### 4.2.8. Bacterial Morphology

Morphological alterations in MRSA cells after exposure to compound **3a** were evaluated using scanning electron microscopy (SEM). A 1 mL bacterial suspension (1 × 10^5^ CFU/mL) was added to a 12-well plate, followed by 1 mL of compound at 2×MIC. Plates were incubated at 37 °C for 24 h. Wells containing only medium served as negative controls.

After incubation, samples were fixed overnight at 4 °C in 3% (*v*/*v*) glutaraldehyde. Samples were dehydrated using a graded ethanol series (50–100%), then coated with gold using a sputter-coating technique. Finally, bacterial morphology was examined by SEM, and structural changes were recorded [53].

#### 4.2.9. Gene Expression

The impact of the synthesized compounds with the lowest IC_50_ values on the expression of *icaA*, *dltB*, *MepA*, 23S rRNA, and other selected target genes in MRSA was examined following previously described procedures [54,55]. After a 24 h treatment period, total RNA was extracted using a commercial isolation kit, and its purity was assessed using a NanoDrop (Wilmington, DE, USA) spectrophotometer (DeNovix DS-11). Complementary DNA (cDNA) was then generated using the Transcriptor First Strand cDNA Synthesis Kit (Roche, Basel, Switzerland). Quantitative real-time PCR (qRT-PCR) analysis was performed using LightCycler^®^ FastStart DNA Master SYBR Green I (Roche). Relative expression levels of the genes were determined based on the 2^−ΔΔCt^ method.

#### 4.2.10. Antioxidant Activity

The antioxidant potential of the **3a**–**3d** was determined by means of the DPPH (2,2-diphenyl-1-picrylhydrazyl) radical scavenging assay, based on a modified protocol of the method first reported by Blois (1958). A 0.10 mM DPPH solution was freshly prepared in methanol prior to analysis and kept protected from light to prevent degradation. **3a**–**3d** compounds at varying concentrations (2, 4, 6, and 8 mg) were introduced into test tubes containing the DPPH solution, followed by thorough mixing using a vortex to ensure uniform dispersion. The reaction mixtures were allowed to stand at ambient temperature for 30 min. Subsequently, absorbance readings were recorded at 517 nm using a UV–Vis spectrophotometer. A DPPH solution without any added sample was considered the negative control, whereas BHT (butylated hydroxytoluene) at concentrations of 0.01–0.04 mg/mL was used as a reference antioxidant. Radical scavenging (%) was calculated relative to the DPPH control, and IC_50_ values were determined from nonlinear regression plots [56].

Similarly, ABTS scavenging activity was determined using a modified spectrophotometric protocol. The ABTS solution was prepared by reacting a 7 mM ABTS solution with 140 mM potassium persulfate, and the resulting mixture was incubated in the dark at room temperature for 16 h to allow complete radical formation. **3a**–**3d** compounds at different concentrations (4, 6, and 8 mg) were mixed with the ABTS solution. The mixtures were homogenized using a vortex mixer and then allowed to react for 30 min at room temperature. Following incubation, the absorbance values were recorded at 734 nm using a UV–Vis spectrophotometer. BHT (0.01–0.04 mg/mL) was used as a positive control standard. The antioxidant activity was calculated relative to the ABTS control [56].

#### 4.2.11. Molecular Docking

Molecular docking studies were performed using Maestro 13.8 (Schrödinger, LLC, New York, NY, USA) to investigate the potential binding modes and interaction profiles of the synthesized compounds with selected target proteins. The 2D chemical structures of all ligands were initially drawn using ChemDraw 15.0 and subsequently converted into their corresponding 3D conformations prior to docking simulations. The crystal structures of the target proteins (PDB IDs: 1MWT, 3VSL, 3ZG5, and 2ZCS) were selected based on their well-documented roles in *Staphylococcus aureus* physiology and pathogenicity. These proteins are associated with critical biological processes such as cell wall biosynthesis, virulence regulation, and antibiotic resistance mechanisms, making them relevant and reliable targets for molecular docking studies aimed at evaluating the antibacterial potential of the synthesized compounds. The crystal structures of the target proteins were retrieved from the Protein Data Bank (PDB). Protein preparation was carried out using the Protein Preparation Wizard, which included the removal of crystallographic water molecules, addition of missing hydrogen atoms, assignment of bond orders, optimization of protonation states, and energy minimization to obtain stable protein structures.

For docking simulations, receptor grids were generated by defining the active site regions of each protein, and the grid box dimensions were set to 20 × 20 × 20 Å. The prepared ligands were docked into the active sites of the target proteins using the Glide XP module, and the binding affinities were calculated. Docking scores were expressed as binding energies in kcal/mol. The best-ranked docking poses were selected based on their docking scores and interaction patterns. Furthermore, key ligand–protein interactions were analyzed in detail to support and rationalize the experimental biological findings [57].

To further validate the docking results, binding free energy calculations were performed using the MM-GBSA (Molecular Mechanics Generalized Born Surface Area) method implemented in the Schrödinger suite 13.8. The calculated ΔG bind values were used to refine the ranking of ligand–protein complexes and to provide additional insight into the stability and strength of the predicted interactions. Consistent trends between docking scores and MM-GBSA results further supported the reliability of the predicted binding modes.

## 5. Conclusions

In this study, a series of Thymol–Benzimidazolium–Chalcone hybrid compounds were successfully synthesized and comprehensively characterized. The synthesized derivatives exhibited notable antioxidant activity, as demonstrated by DPPH and ABTS assays. In addition, all compounds showed measurable antibacterial effects against clinical *Staphylococcus aureus* isolates, with compound **3a** emerging as the most potent candidate based on MIC, MBC, and disk diffusion results. Mechanistic investigations revealed that compound **3a** caused significant morphological alterations in bacterial cells, as confirmed by SEM analysis, suggesting disruption of cell integrity. Furthermore, gene expression analysis demonstrated that compound **3a** effectively downregulated key genes associated with biofilm formation and bacterial resistance, namely *icaA*, *dltB*, and *mepA*, indicating a multi-target mode of action. Notably, the pronounced suppression of *dltB* highlights its potential role in compromising cell wall stability and enhancing antibacterial susceptibility. Molecular docking studies supported the experimental findings, showing favorable binding affinities of the synthesized compounds toward selected bacterial target proteins, with compound **3a** exhibiting the strongest interaction, particularly with the 2ZCS protein. These results suggest that the observed biological activities are consistent with the predicted ligand–protein interactions.

Overall, the integration of experimental and in silico analyses indicates that compound **3a** represents a promising lead structure with significant antibacterial, antibiofilm, and antioxidant potential. However, further studies, including in vivo evaluations and detailed mechanistic investigations, are required to fully elucidate its therapeutic applicability and safety profile.

## Figures and Tables

**Table 1:** The MIC and MBC values (µg/mL) of the compounds against clinical MRSA strains.

Bacterial Strain	3a		3b		3c		3d		Vancomycin	
	MIC	MBC	MIC	MBC	MIC	MBC	MIC	MBC	MIC	MBC
MRSA 568	1	4	1	2	2	8	1	32	2	4
MRSA 565	1	4	0.5	1	2	32	1	64	2	4
MRSA 553	0.5	0.5	0.5	1	4	128	4	128	2	4
MRSA 552	0.5	0.5	4	4	8	128	8	128	2	4
MRSA 551	0.5	2	1	1	4	64	1	64	2	4
MRSA 540	0.5	0.5	0.5	4	8	128	4	64	2	4
MRSA 539	0.25	0.5	0.25	0.5	2	128	1	32	2	4
MRSA 529	0.5	0.5	1	2	8	128	1	32	2	4

**Table 2:** IC_50_ (µg/mL) values of **3a**–**3d** compounds against DPPH and ABTS.

Compounds	DPPH	ABTS
**3a**	73.1 ± 0.34	67.1 ± 0.72
**3b**	148.1 ± 0.67	27.5 ± 0.43
**3c**	97.5 ± 0.54	92.8 ± 0.82
**3d**	140 ± 0.71	73.4 ± 0.7
**BHT**	23.4 ± 0.42	26.3 ± 0.63

**Table 3:** Molecular docking scores, glide emodel values and MMGBSA of **3a**.

Protein	Docking Score (kcal/mol)	Glide e Model	MMGBSA
1MWT	−5.310	−67.712	−52.81
3VSL	−6.145	−70.223	−63.31
3ZG5	−7.043	−75.749	−66.20
2ZCS	−11.953	−87.034	−58.77

## Data Availability

The datasets used and/or analyzed during the current study are available from the corresponding author upon reasonable request.

## Supplementary Materials

The following supporting information can be downloaded at: https://www.mdpi.com/article/10.3390/antibiotics15050477/s1.
