# Supplementary material for: Antibacterial and Antibiofilm Potential of Thymol–Benzimidazolium–Chalcone Hybrids Against Clinical MRSA Strains: Insights from Gene Expression Profiling and Molecular Docking
Source: Antibiotics (Basel). 2026 May 8;15(5):477. doi: 10.3390/antibiotics15050477 (PMC13203663; doi:10.3390/antibiotics15050477)
Supplement: Supplementary file 1 [file antibiotics-15-00477-s001.zip › antibiotics-4261794-supplementary.pdf]

Supporting Information

Antibacterial and Antibiofilm Potential of Thymol–Benzimidazolium–Chalcone Hybrids Against Clinical MRSA Strains: Insights from Gene Expression Profiling and Molecular Docking

Salim Yakut[1], Hakan Ünver[2], Akın Yiğin[3], Mehmet Çimentepe[4], Fadile Yıldız Zeyrek[1], Özge Öztürk Çimentepe[5], Metin Yildirim[6]

[1]Department of Medical Microbiology, Faculty of Medicine, Harran University, Sanliurfa, TR-63200, Türkiye

[2]Department of Chemistry, Faculty of Science, Eskisehir Technical University, Eskisehir, Türkiye

[3]Department of Genetics, Faculty of Veterinary Medicine, Harran University, Sanliurfa, TR-63200, Türkiye

[4]Department of Pharmaceutical Microbiology, Faculty of Pharmacy, Harran University, Sanliurfa, TR-63200, Türkiye

[5]Department of Pharmacology, Faculty of Pharmacy, Harran University, Sanliurfa, TR-63200, Türkiye

[6]Department of Biochemistry, Faculty of Pharmacy, Cukurova University, Adana, TR-01330, Türkiye

---

*To whom correspondence should be addressed: Cukurova University, Faculty of Pharmacy, Department of Biochemistry, Adana, Türkiye. e-mail: metinyildirim4@gmail.com; http://orcid.org/0000-0003-1346-312X **(M. Yildirim)**;

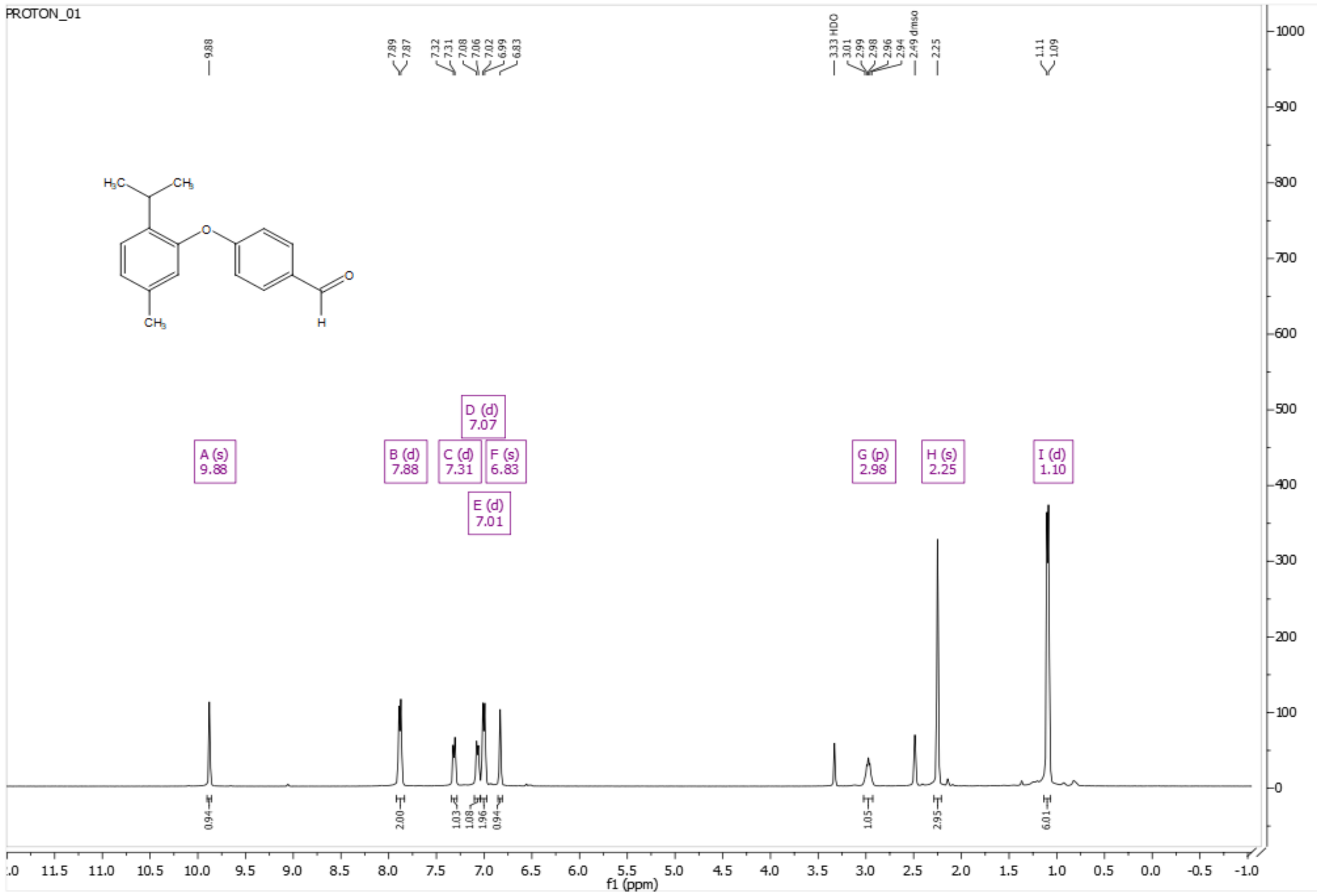

Figure S1: Compound 1 ^{1}H-NMR spectra

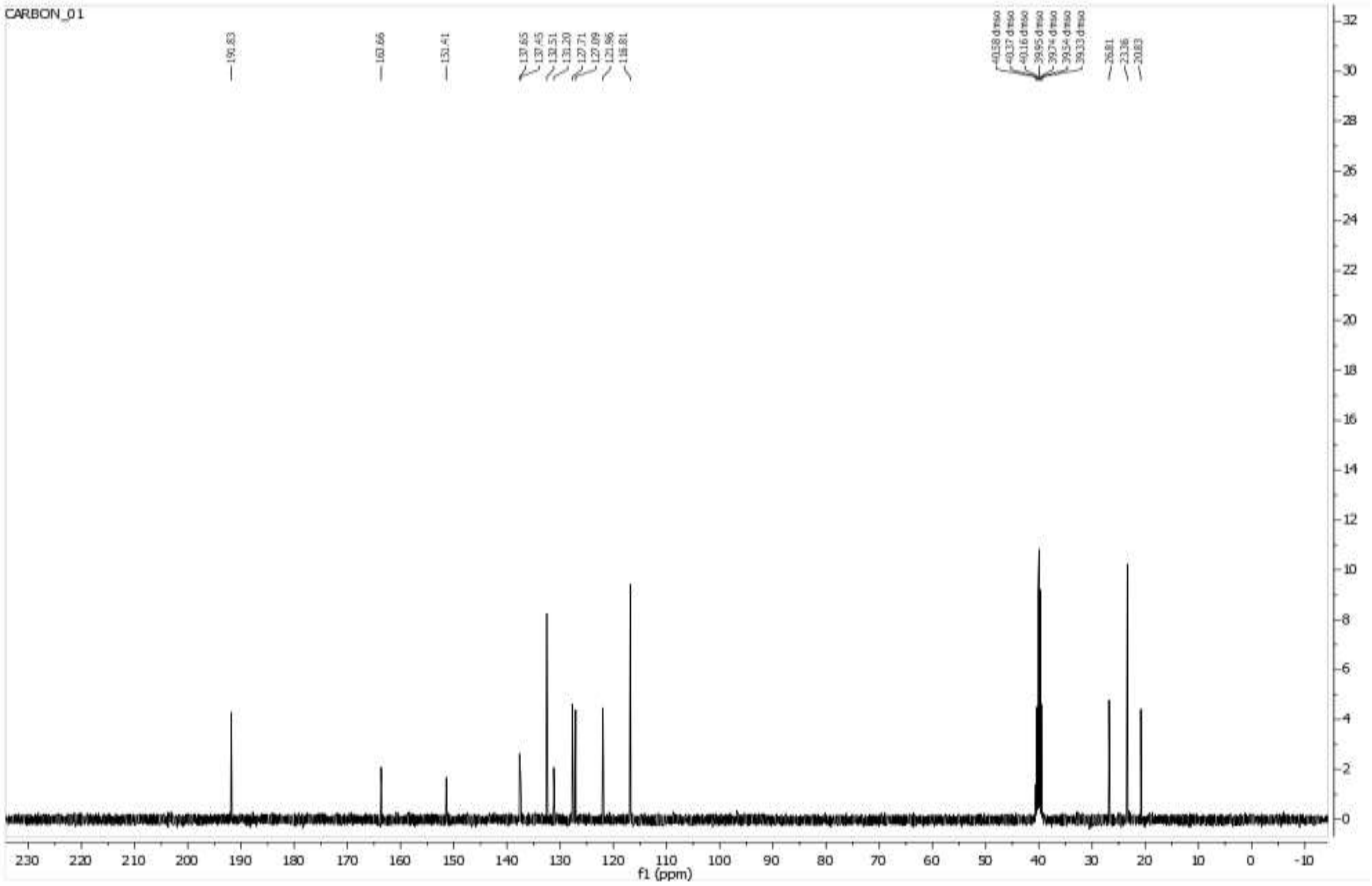

Figure S2: Compound 1 ^{13}C-NMR spectra

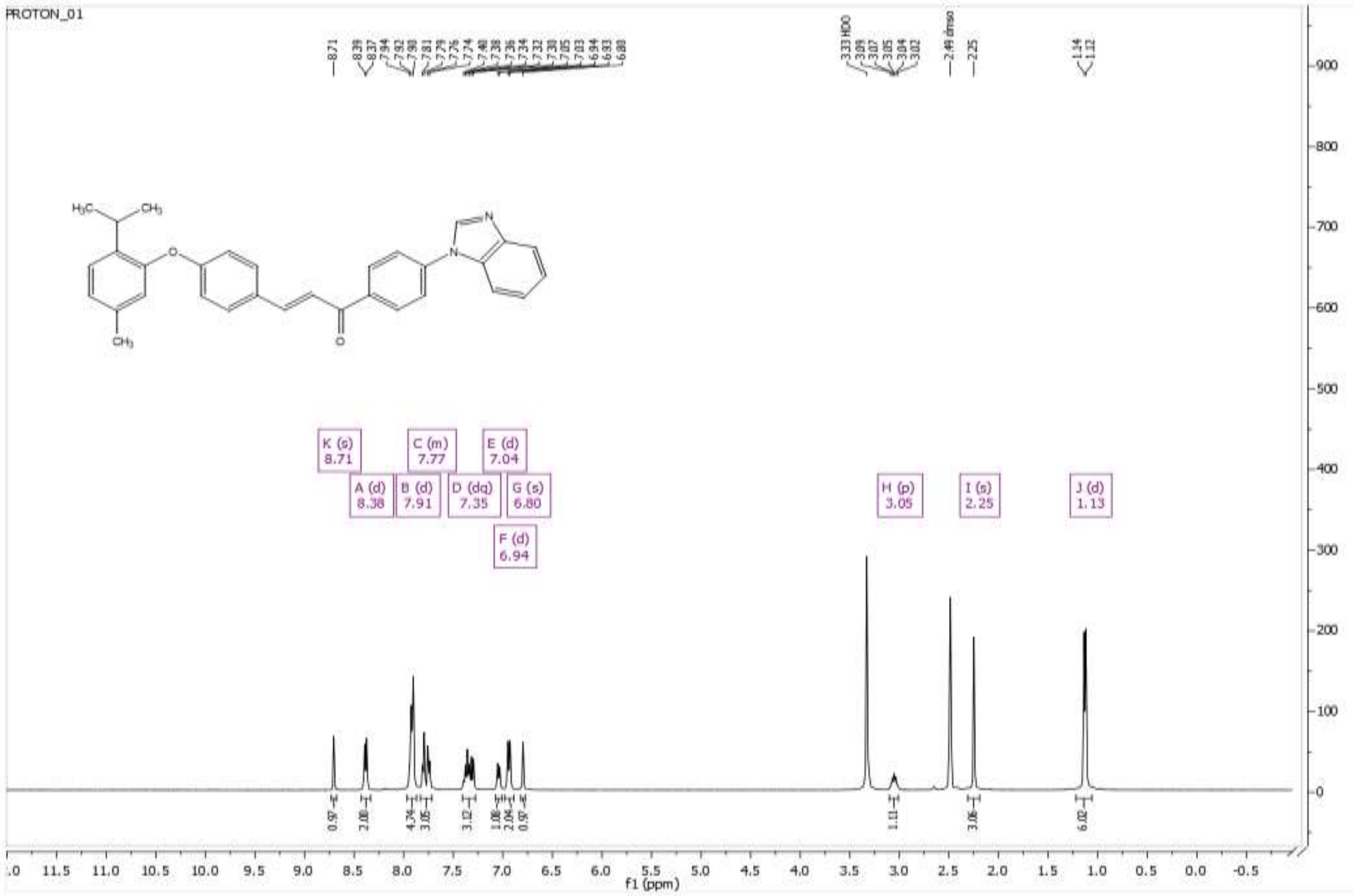

Figure S3: Compound 2 ^{1}H-NMR spectra

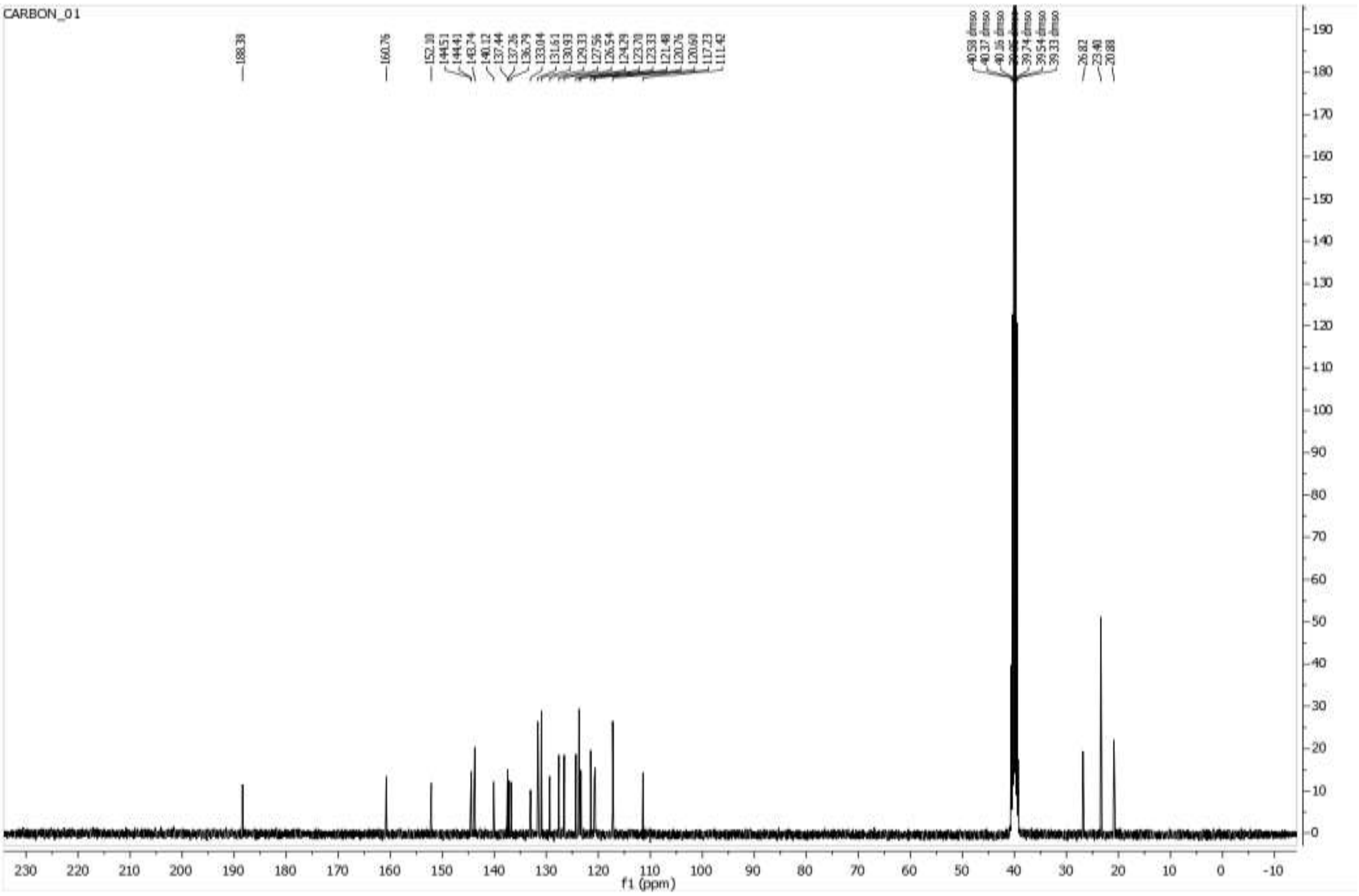

Figure S4: Compound 2 ^{13}C-NMR spectra

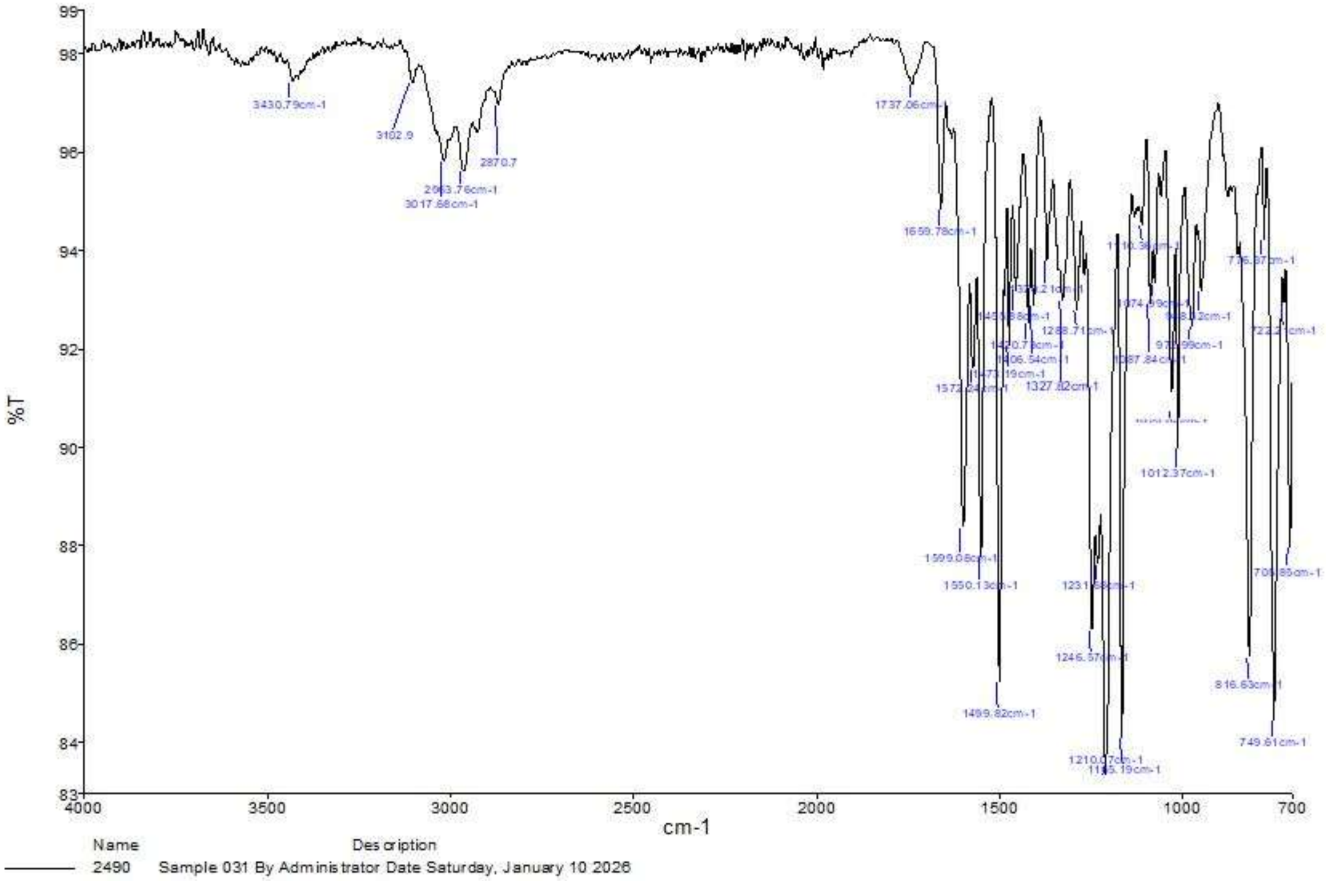

Figure S5: Compound 3a FT-IR spectra

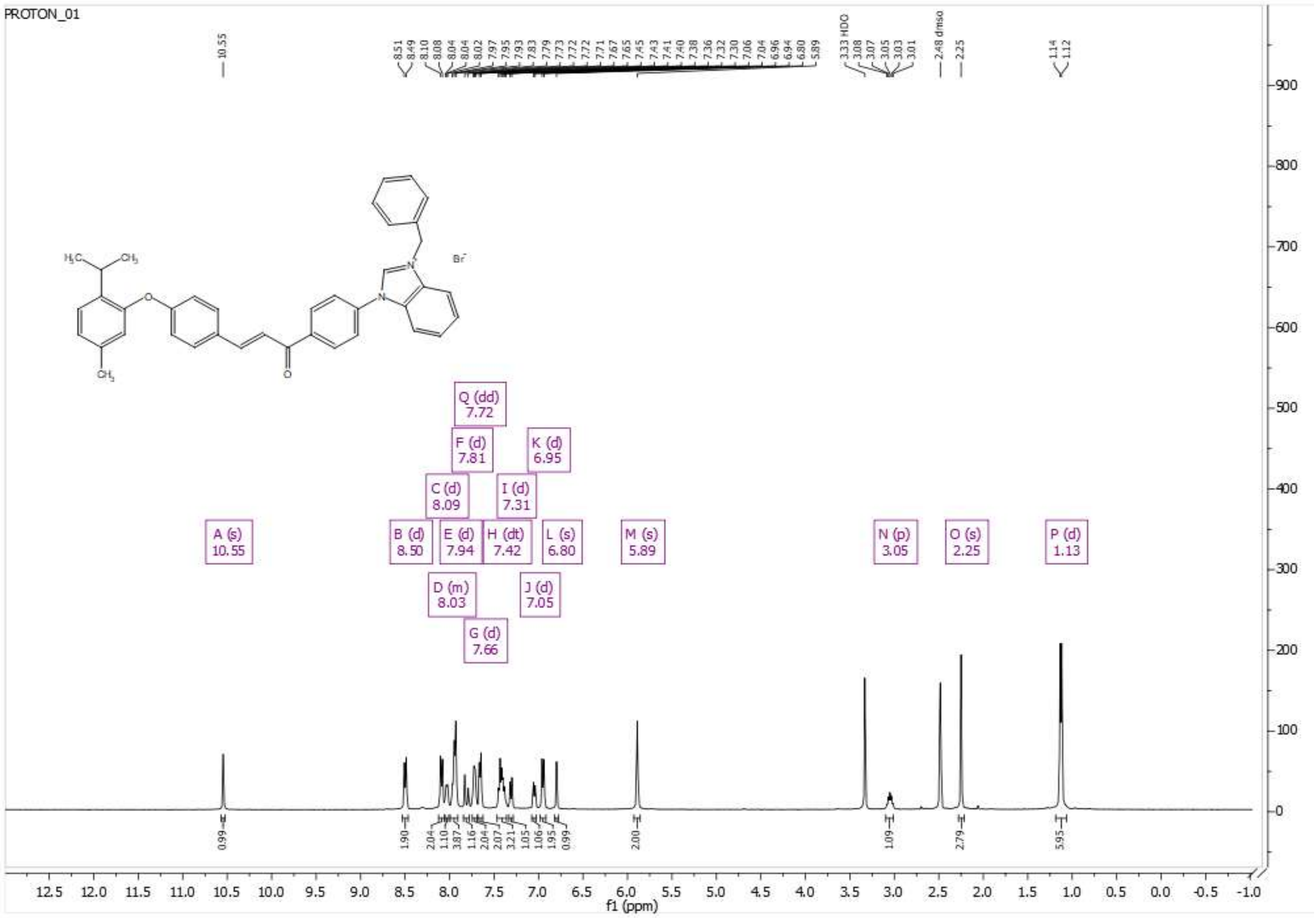

Figure S6: Compound 3a ^{1}H-NMR spectra

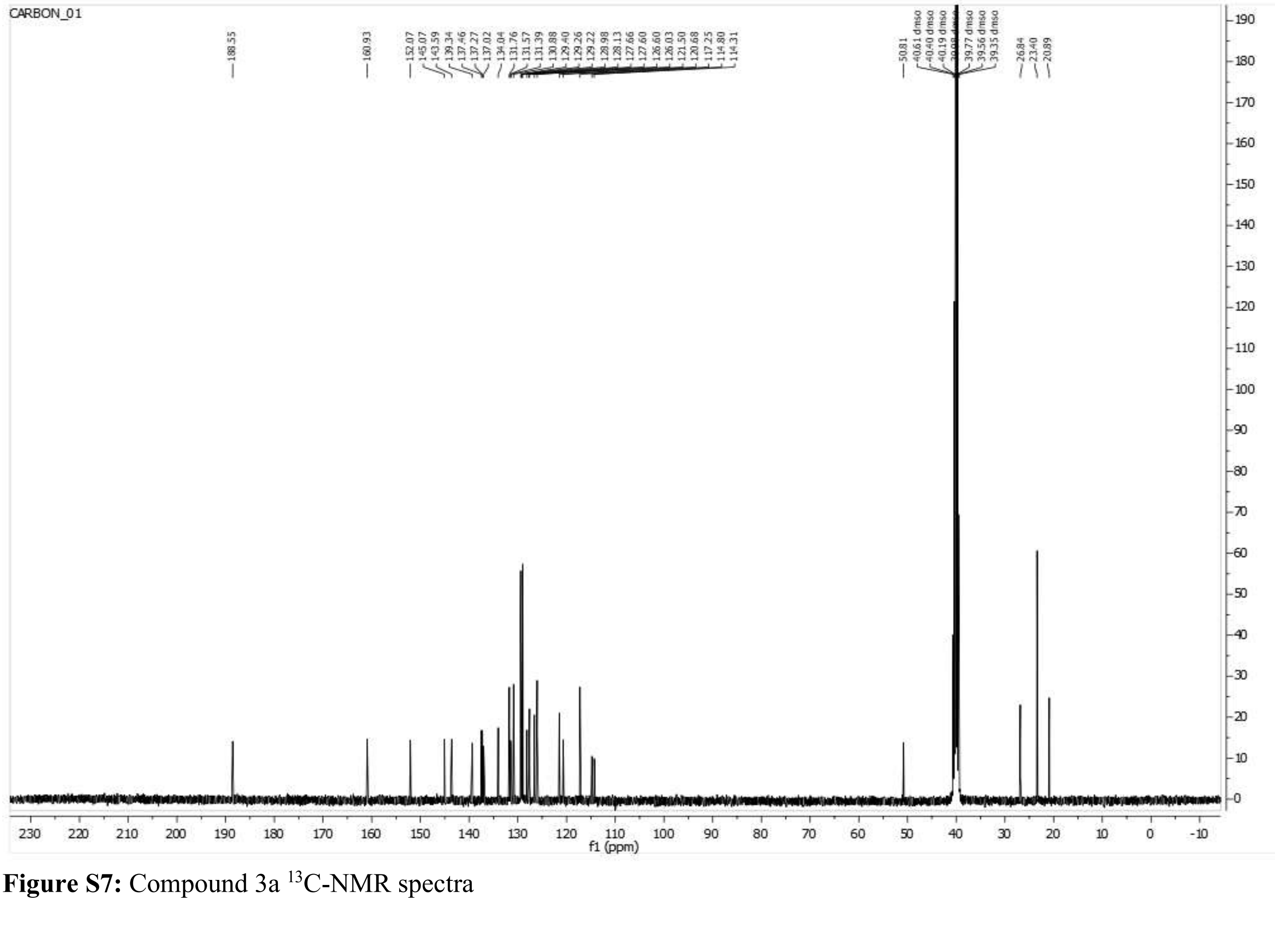

Figure S7: Compound 3a ^{13}C-NMR spectra

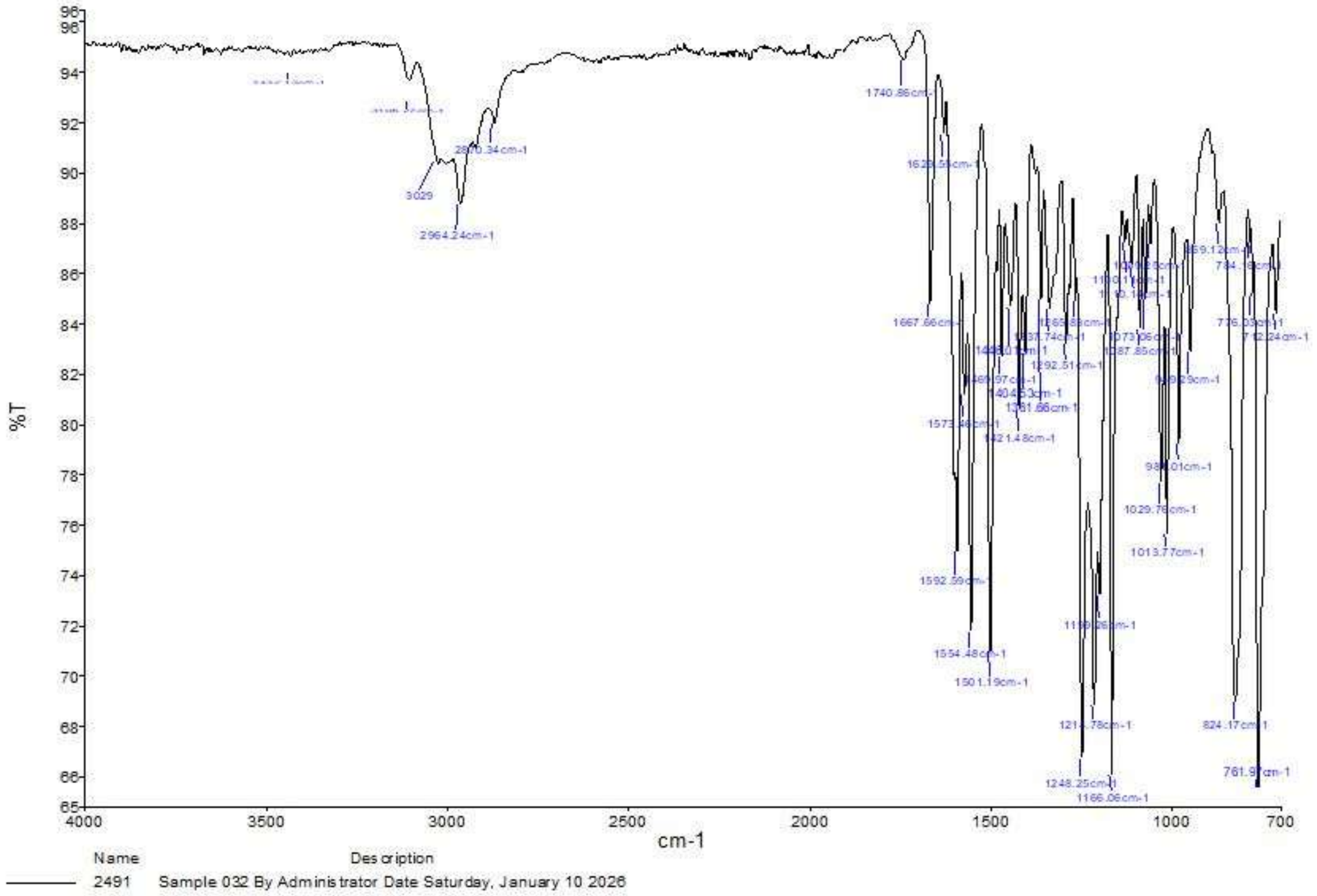

Figure S8: Compound 3b FT-IR spectra

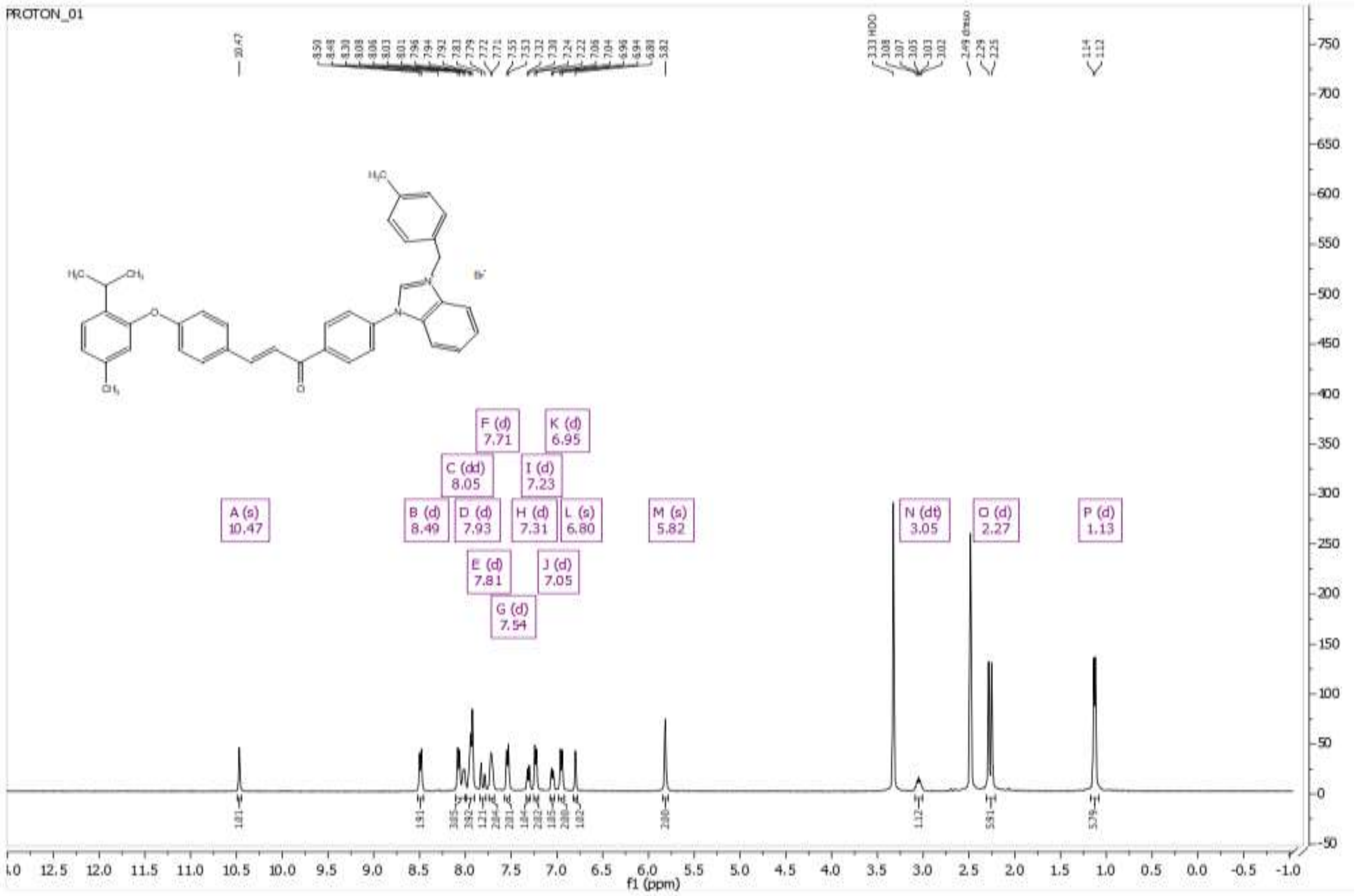

Figure S9: Compound 3b ^{1}H-NMR spectra

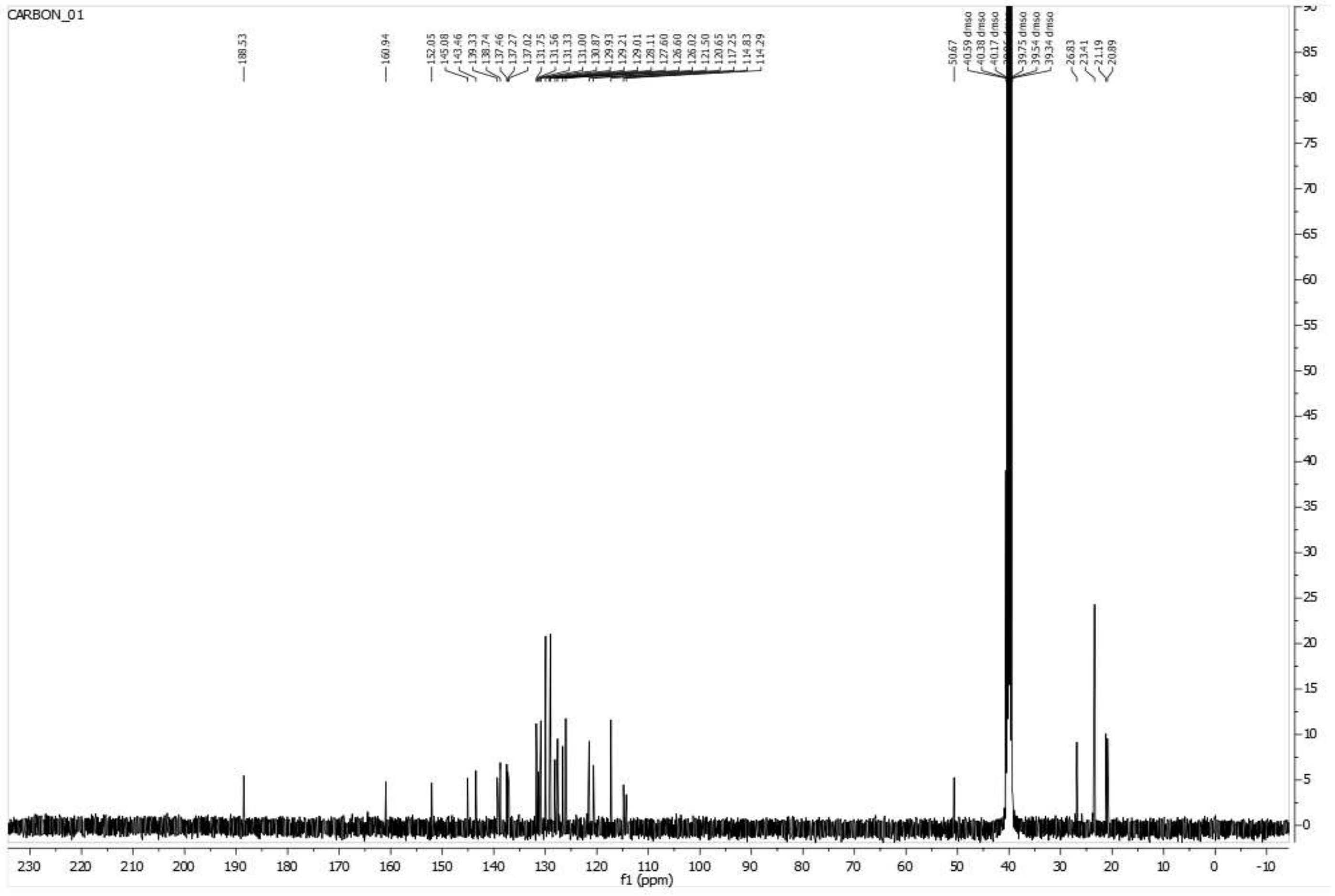

Figure S10: Compound 3b ^{13}C-NMR spectra

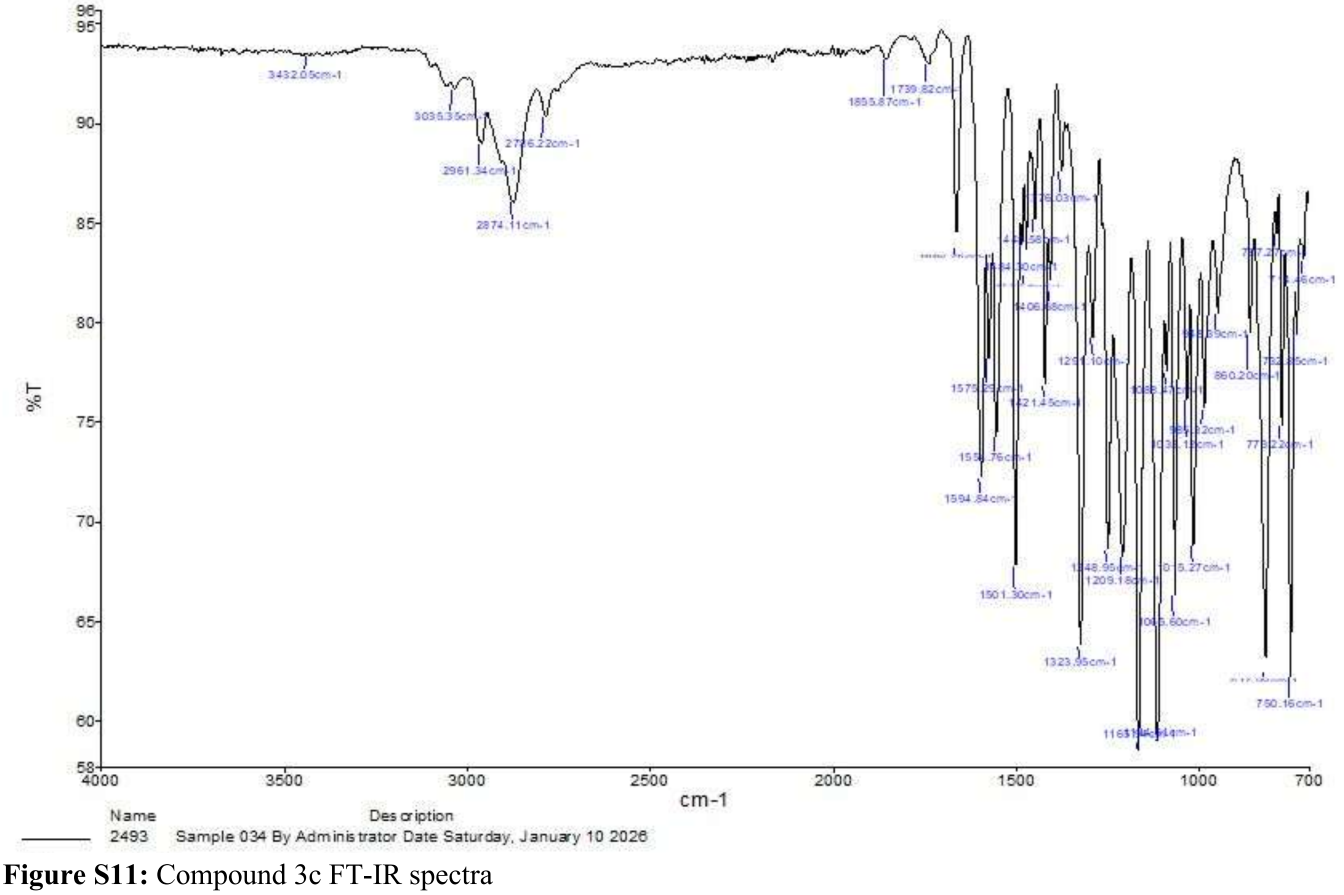

Figure S11: Compound 3c FT-IR spectra

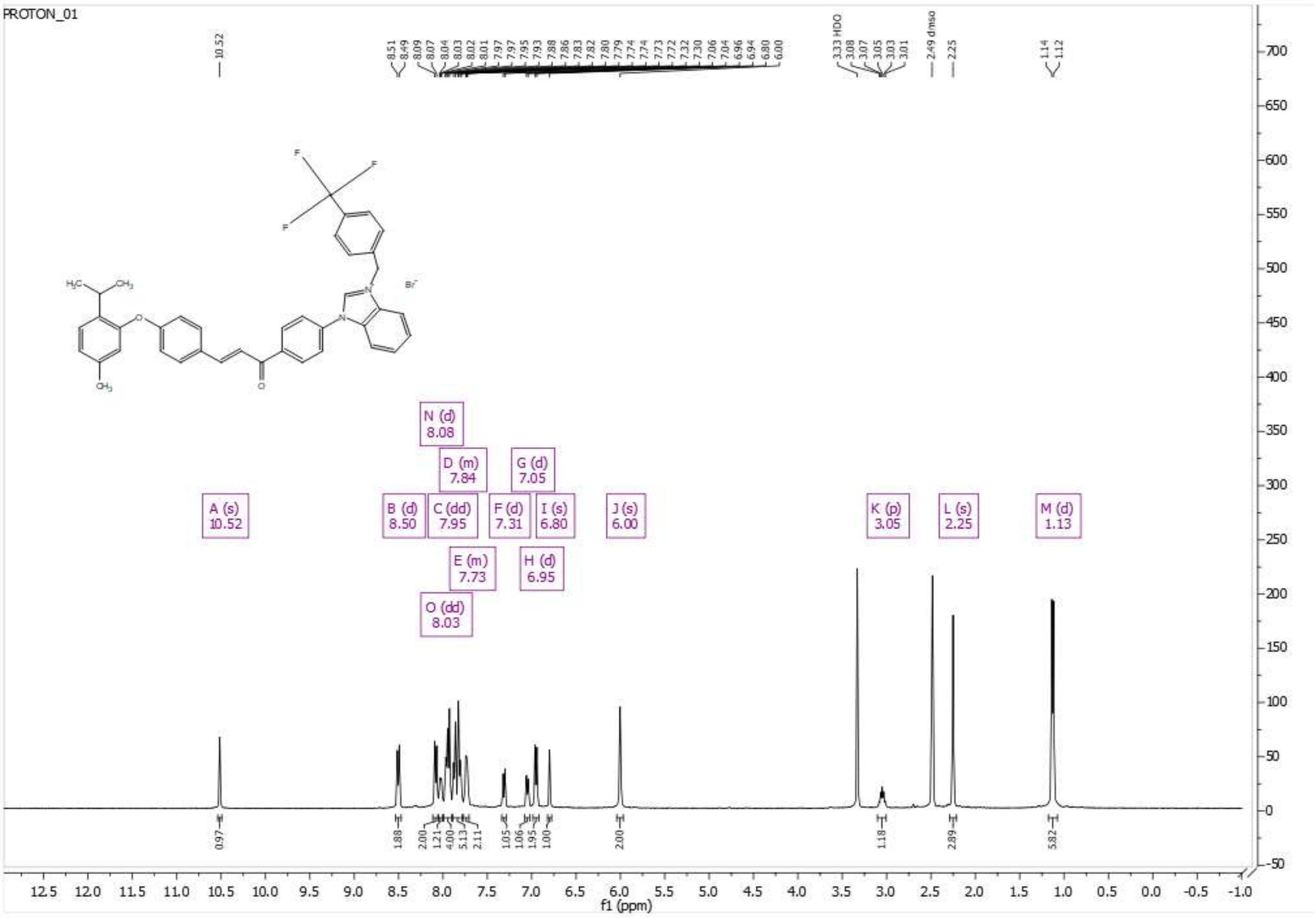

Figure S12: Compound 3c ^{1}H-NMR spectra

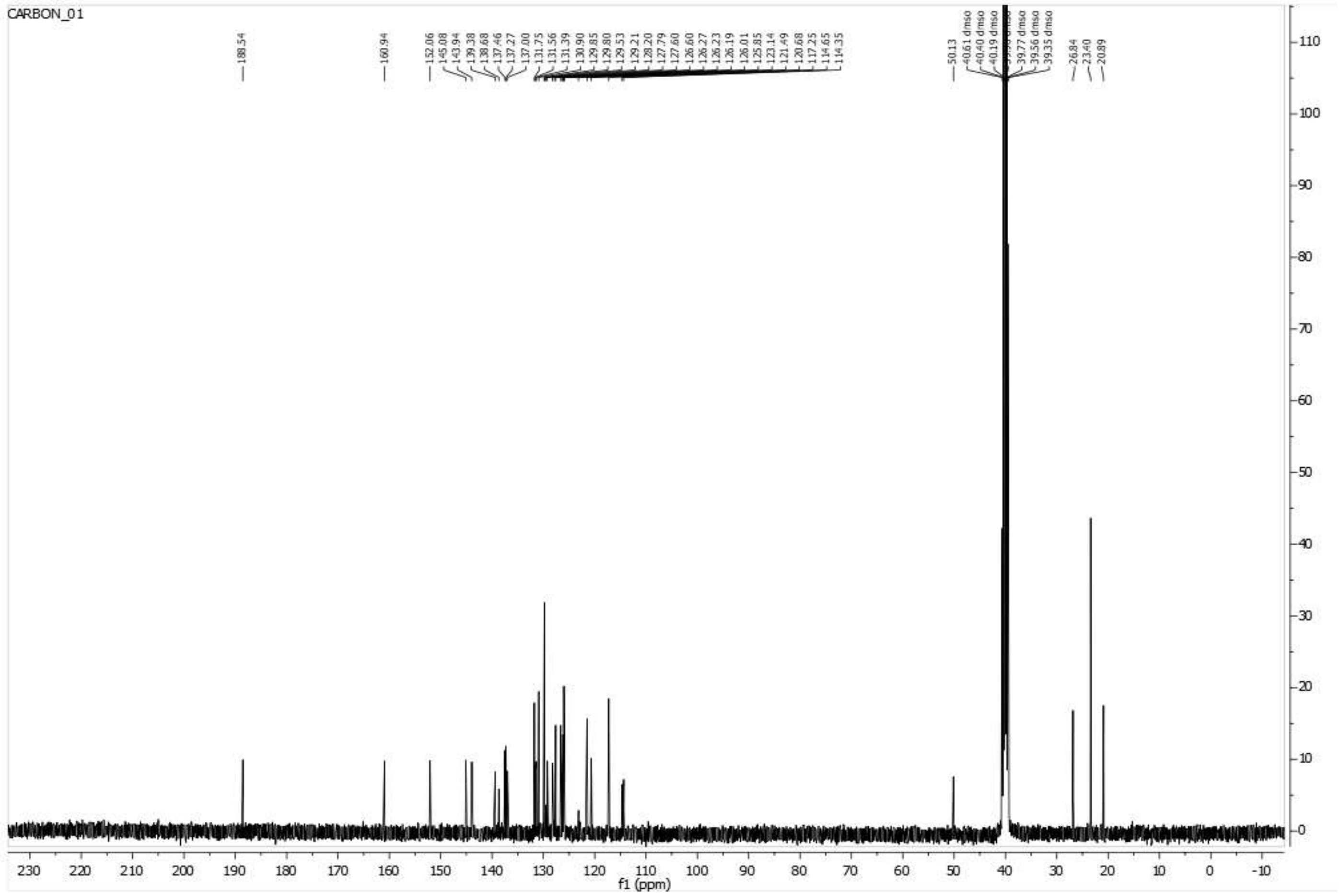

Figure S13: Compound 3c ^{13}C-NMR spectra

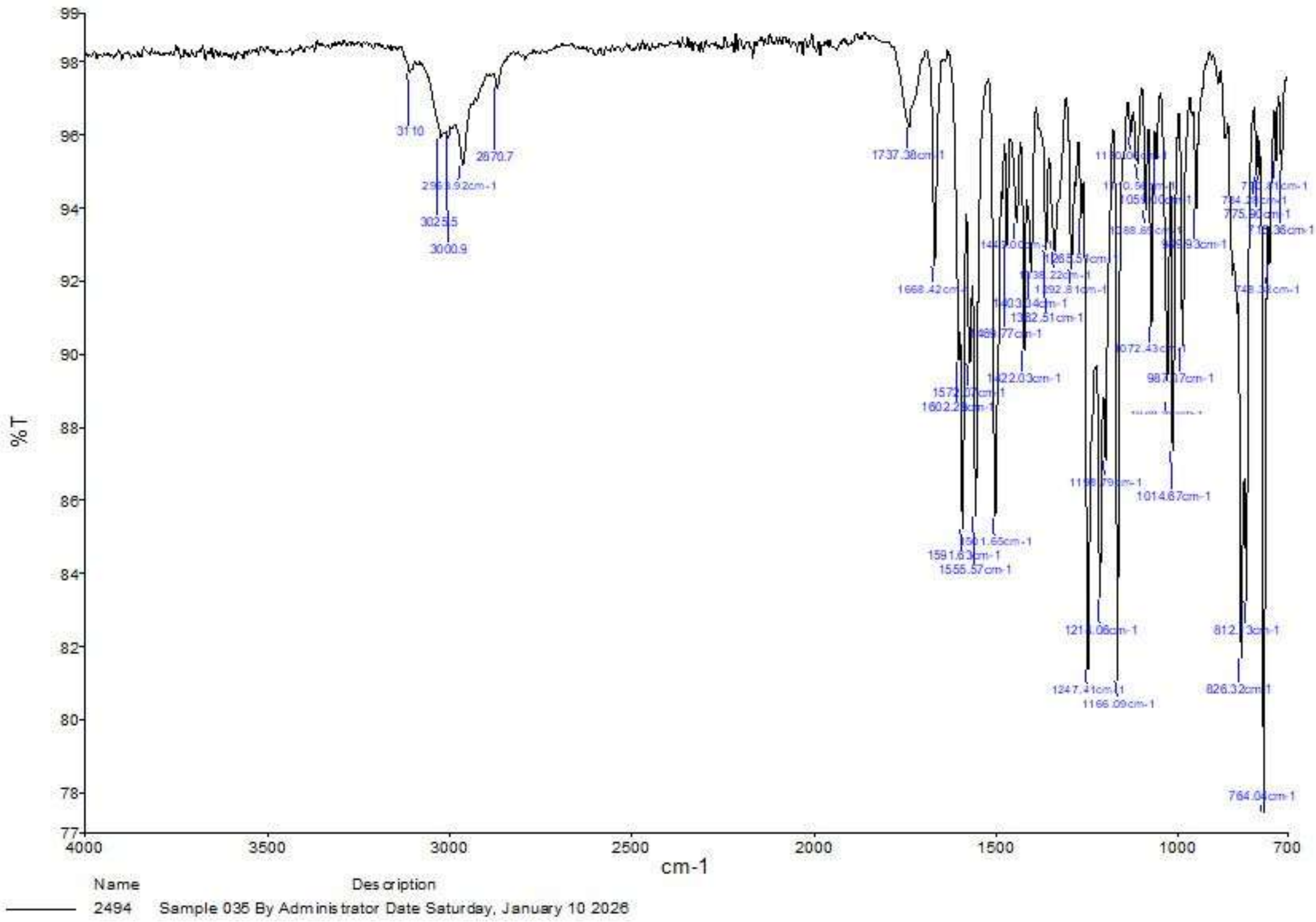

Figure S14: Compound 3d FT-IR spectra

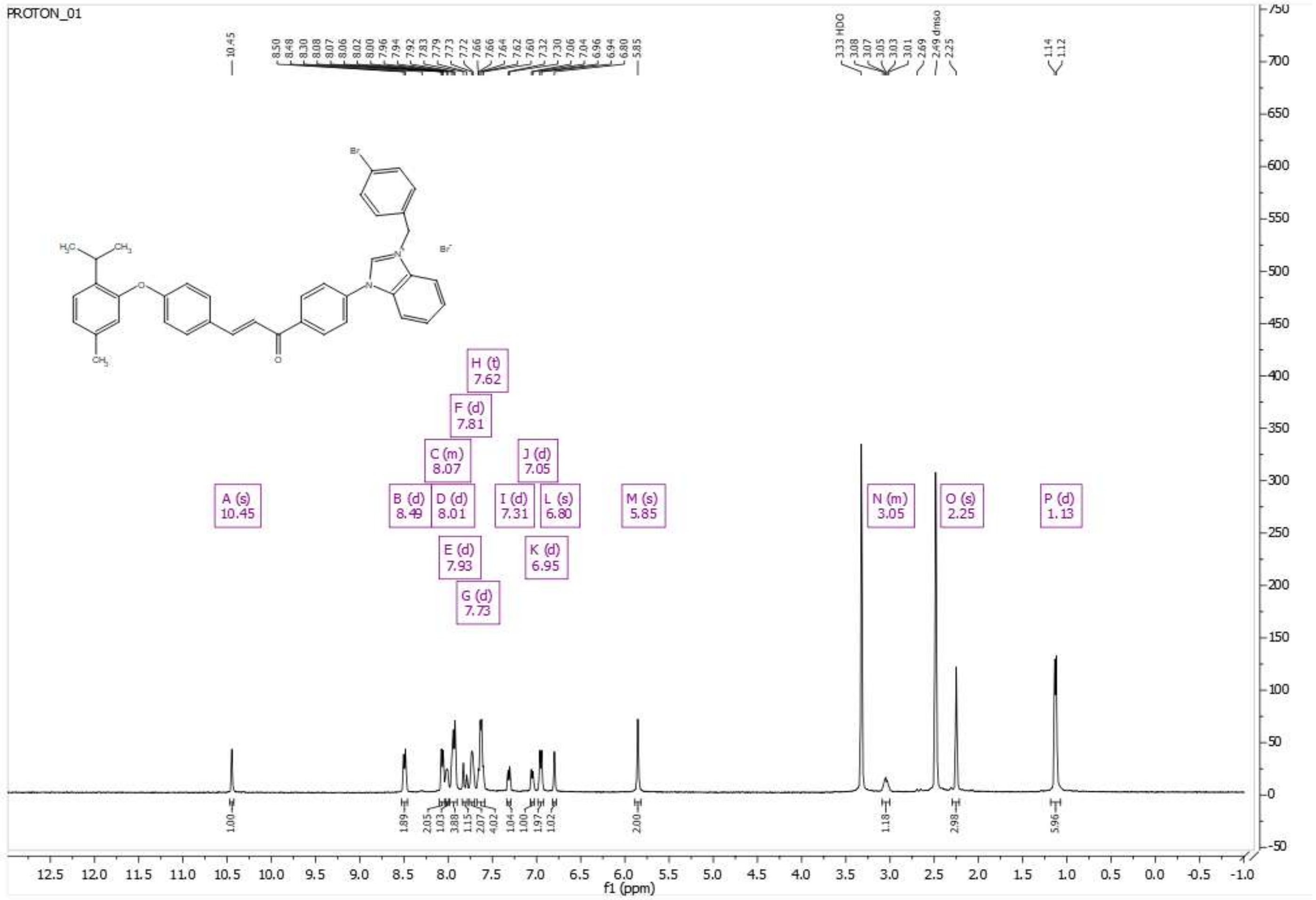

Figure S15: Compound 3d ^{1}H-NMR spectra

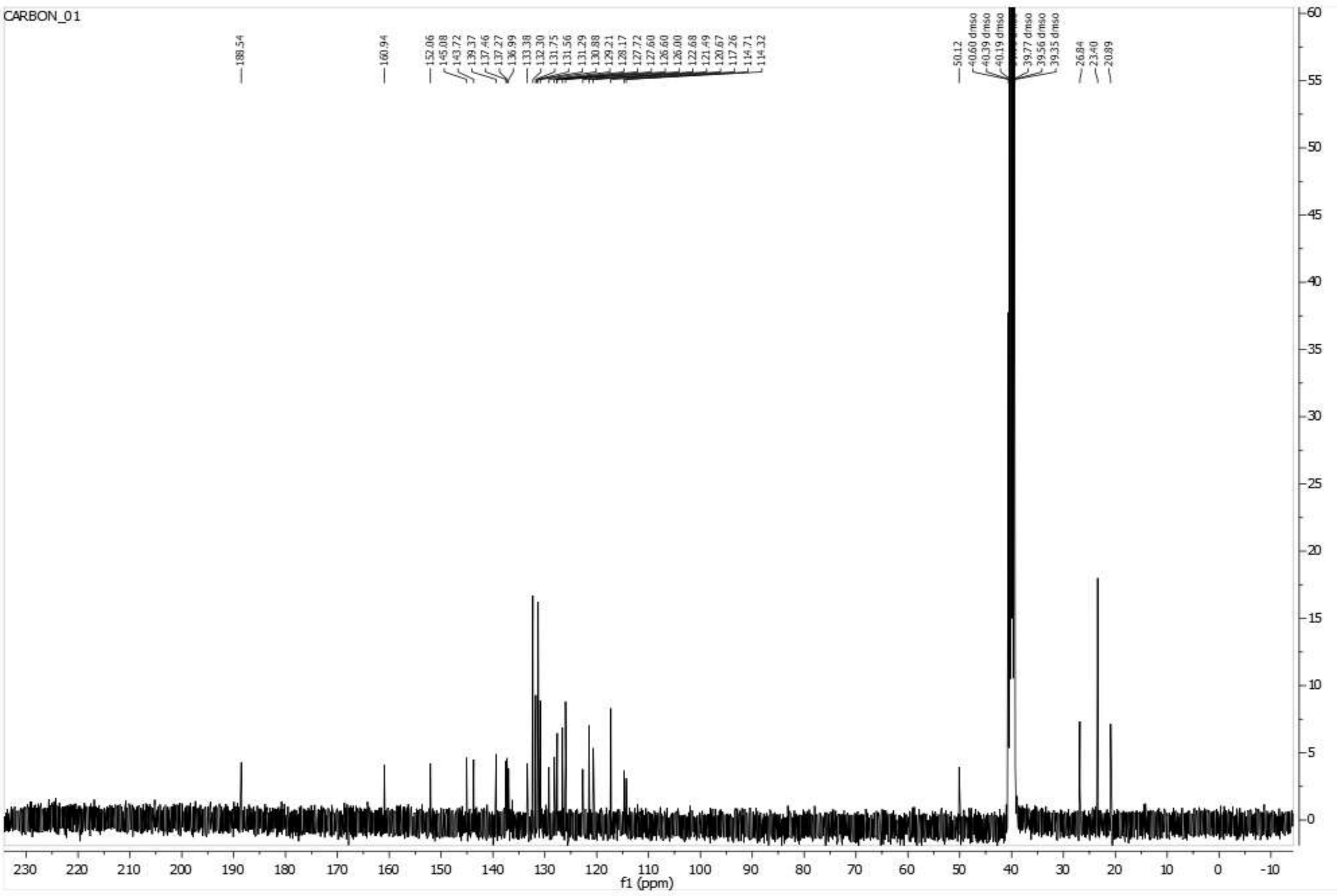

Figure S16: Compound 3d ^{13}C-NMR spectra

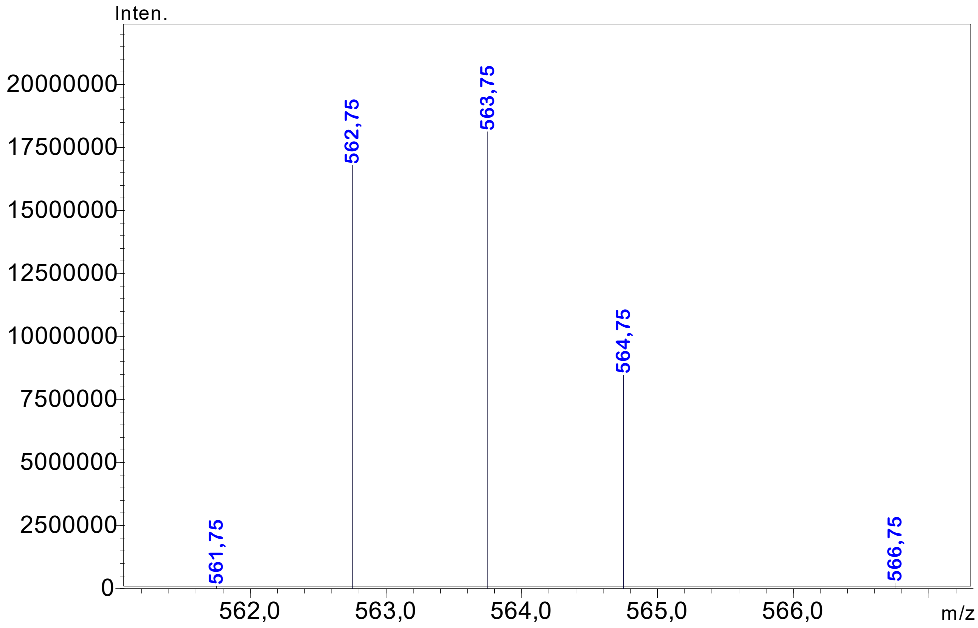

Figure S17. LC-MS/MS spectra of the 3a compound

3a

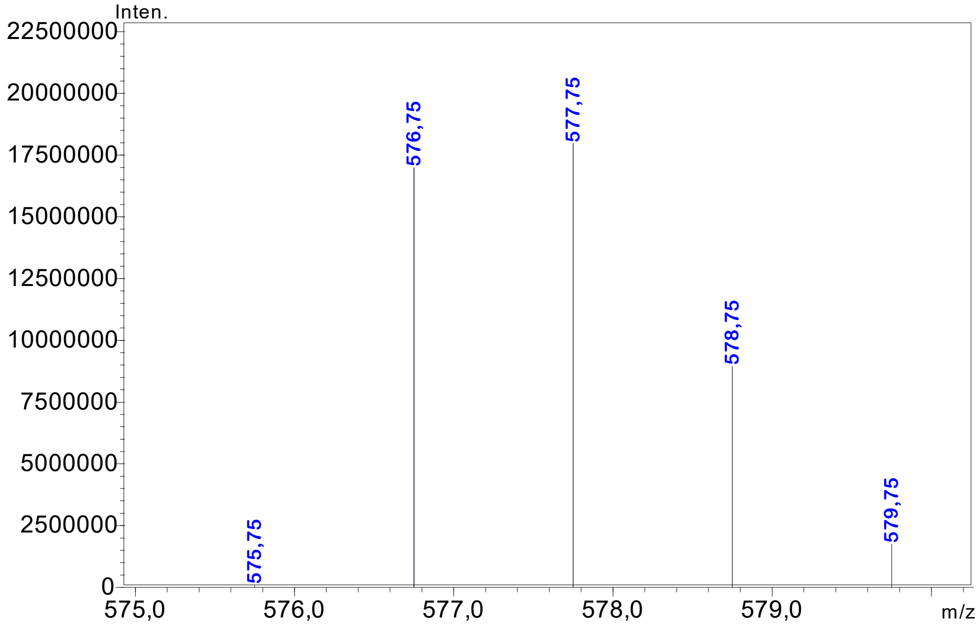

Figure S18. LC-MS/MS spectra of the 3b compound

2491

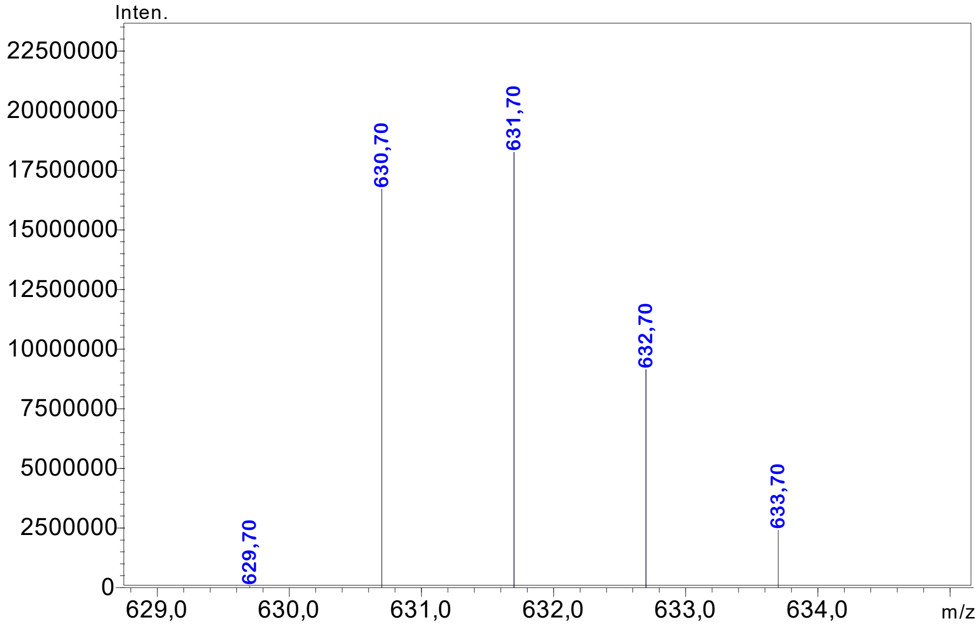

Figure S19. LC-MS/MS spectra of the 3c compound

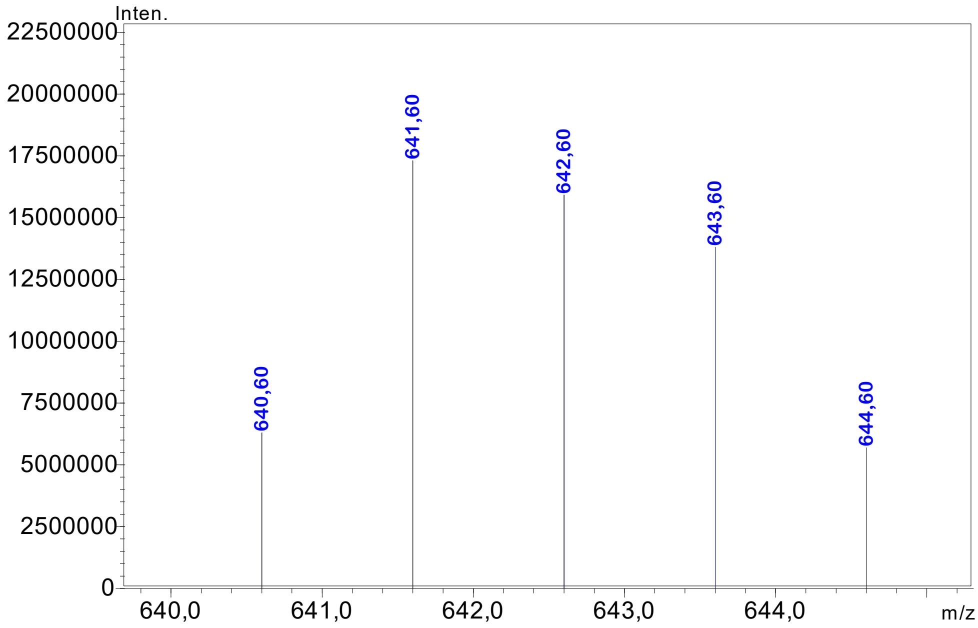

Figure S20. LC-MS/MS spectra of the 3d compound
